# Forecasting supply and demand in nursing professions: impacts of occupational flexibility and employment structure in Germany

**DOI:** 10.1186/1478-4491-11-24

**Published:** 2013-06-05

**Authors:** Tobias Maier, Anja Afentakis

**Affiliations:** 1 Section ‘Qualifications, Occupational Integration and Employment’, Federal Institute for Vocational Education and Training, Robert-Schuman-Platz 3, 53175, Bonn, Germany; 2 Section ‘Health Accounting Systems’, Federal Statistical Office, Graurheindorfer Str. 198, 53117, Bonn, Germany

**Keywords:** Forecast for nursing professions, Occupational flexibility, Employment structure, Labour Force Survey

## Abstract

**Background:**

In light of Germany's ageing society, demand for nursing professionals is expected to increase in the coming years. This will pose a challenge for policy makers to increase the supply of nursing professionals.

**Methodology:**

To portray the different possible developments in the supply of nursing professionals, we projected the supply of formally trained nurses and the potential supply of persons who are able to work in a nursing profession. This potential supply of nursing professionals was calculated on the basis of empirical information on occupational mobility provided by the German Microcensus 2005 (Labour Force Survey). We also calculated how the supply of full-time equivalents (FTEs) will develop if current employment structures develop in the direction of employment behaviour in nursing professions in eastern and western Germany. We then compared these different supply scenarios with two demand projections ('status quo' and 'compression of morbidity' scenarios) from Germany's Federal Statistical Office.

**Results:**

Our results show that, even as early as 2005, meeting demand for FTEs in nursing professions was not arithmetically possible when only persons with formal qualification in a nursing profession were taken into account on the supply side. When additional semi-skilled nursing professionals are included in the calculation, a shortage of labour in nursing professions can be expected in 2018 when the employment structure for all nursing professionals remains the same as the employment structure seen in Germany in 2005 (demand: 'status quo scenario'). Furthermore, given an employment structure as in eastern Germany, where more nursing professionals work on a full-time basis with longer working hours, a theoretical shortage of nursing professionals could be delayed until 2024.

**Conclusions:**

Our analysis of occupational flexibility in the nursing field indicates that additional potential supply could be generated by especially training more young people for a nursing profession as they tend to stay in their initial occupation. Furthermore, the number of FTEs in nursing professions could be increased by promoting more full-time contracts in Western Germany. Additionally, employment contracts for just a small number of weekly working hours (marginal employment) cannot be considered an adequate instrument for keeping formally trained nursing professionals employed in the nursing field.

## Background

The care of sick and elderly people is personnel-intensive and must be carried out primarily by formally trained persons. This is an area where Germany with its shrinking and ageing population will soon reach its limits [1]. In this paper, we focus on nursing professions as defined by the German Classification of Occupation 1992 (KldB 92). This includes nurses and midwives (Category 853, KldB 92), auxiliary nurses (Category 854) and geriatric nurses (Category 864). In Germany, graduated nurses or geriatric nurses have 3 years of formal training and are able to conduct all nursing duties. In contrast, an auxiliary (geriatric) nurse has only a one- to two-year training programme and thus, is given a restricted list of tasks, compared to nurses and midwives and geriatric nurses [2]. Unfortunately, the KldB 92 does not differentiate between geriatric and auxiliary geriatric nurses. Therefore, all individuals with a vocational training of at least one year duration within these three groups will be referred to as formally trained nursing professionals in the following. It is important to remember that vocational training for graduate nurses and graduate geriatric nurses is highly regulated in Germany. However, since the curricula for these two occupations are similar, geriatric nurses can, in practice, be employed as regular nurses and vice versa. Nonetheless, this always depends on the particular duties to be performed.

The situation is different at the level of auxiliary (geriatric) nurses. Similar, for example, to the US [3] or UK [2], in Germany it is also possible for an individual to perform certain nursing duties such as performed by (geriatric) nursing assistants after having received a few weeks or months of corresponding training. In the following, such individuals who have received less than one year of training will be referred to as semi-skilled labour nurses. As for other occupational classifications [4,5], the KldB 92 classifies a set of tasks without a strong distinction between the different formal qualifications required to fulfil those tasks. This means that (geriatric) nursing assistants with less than one year of training are classified as being employed as auxiliary nurses (category 854) or geriatric nurses (category 864) (see endnotes for more detailed information).

Forecasting models for the supply of and demand for nurses usually concentrate only on the inflow of graduates and/or immigrants from other countries and on the attrition and retirement rates for graduate nurses [6]. However, semi-skilled nursing professionals can improve organizational efficiency [7] and will be needed to meet the increasing demand for care. In Germany, the number of auxiliary nurses has steadily increased from 209 000 persons in 2000 to 269 000 persons in 2010 [8]. Since the number of employed semi-skilled labour nurses is mainly contained in the number of auxiliary nurses, these developments indicate that semi-skilled labour is becoming increasingly important for meeting the demand for nurses. Consequently, in order to forecast the supply of nursing professionals, we have to examine both the occupational outflows of formally trained nursing professionals [9] and inflows of persons with less than one year of training, that is, occupational mobility into and out of nursing professions (Additional file 1).

For this paper, we analyzed occupational flexibility and different employment structures (full-time, part-time and number of hours worked per week) to identify ways to increase the supply of nursing professionals. We then contrasted our different supply scenarios with two demand scenarios of nursing professionals (nurses and midwives, auxiliary nurses and geriatric nurses) developed by the German Federal Statistical Office [1].

## Methodology

### Variables and data sets

Whether the supply of nursing professionals will be able to meet demand in the future depends on occupational flexibility and employment structures in the nursing field. The term employment structure refers to the proportion of full-time, part-time and marginal part-time workers, that is, persons who earn under 400 euros a month and whose earnings are not subject to social security contributions (*geringfügige Beschäftigung*), and the number of weekly hours they work. It is possible to render different employment structures comparable by calculating full-time equivalents (FTEs). This is why FTEs – rather than headcounts – were used to calculate the supply and demand forecasts for nursing professionals.

**Figure 1:**
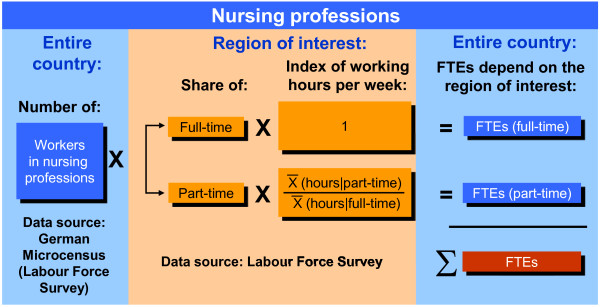
**Full-time equivalents (FTEs) in nursing professions, by employment structure of the region of interest.**

Nursing professionals work primarily in hospitals, ambulatory nursing care and (semi-) stationary nursing homes (daytime nursing care facilities). According to the Health Personnel Accounting system, these three types of health care providers accounted for 76.3% of all FTEs in nursing professions in the year 2005 and will be referred to below as health care providers. The unit of measure for presentation of the following results is the number of FTEs in nursing professions at health care providers, since they can be calculated as part of the Health Personnel Accounting system. We used the Microcensus to apply the employment structure for nursing professions of eastern and western Germany to all nursing professions (see Figure 1 for the method). The German Labour Force Survey (LFS) is an integral part of the German Microcensus and also provides this information. To enable the use of this concept in other countries, we have provided the requisite information from the European Union Labour Force Survey in Table 1 (for the calculation of FTEs, please see the variables FTPT and HWUSUAL).

**Table 1:** Commission Regulation (EC) No 377/2008 of 25 April 2008 (abridgment of codification)

**Name**	**Description**
**ISCO4D**	Occupation: ISCO08, coded at the 3- or if possible 4-digit level
**HATLEVEL**	Highest level of education or training successfully completed: Classification of Education (ISCED), coded at the 2-digit level
**HATFIELD**	Field of highest level of education or training successfully completed: Classification of Education Field (ISCED field), coded at the 1-digit level
**FTPT**	Full-time/part-time distinction:
	1 = full-time
	2 = part-time
**HWUSUAL**	Number of hours per week usually worked in the main job

In contrast to the current occupation that respondents are directly asked to provide in the Microcensus (for the EU LFS, see ISCO4D in Table 1), the initial vocational qualification of the economically active population has to be reconstructed heuristically by combining the qualification level (highest level achieved according to the International Standard Classification of Education, ISCED) with the major field of study [10] (for the EU LFS, see HATLEVEL and HATFIELD in Table 1), in other words:

•(Qualification level: ISCED 5b) + (major field of study: nursing) = (Category 853, KldB 92: nurses and midwives).

•(Qualification level: ISCED 3b/4) + (major field of study: nursing or rescue service) = (Category 854, KldB 92: auxiliary nurses).

•(Qualification level: ISCED 5b) + (major field of study: geriatric care) = (Category 864, KldB 92: geriatric nurses).

This method of determining the initial vocational qualification requires a profound knowledge of the (German) education system because Microcensus respondents have to classify themselves and some fields of study, for example, are available only at the academic level and not at the intermediate skill level (ISCED 3b/4). Implausible combinations were removed from the analysis. However, as the major field of study is only surveyed with the highest qualification achieved, it cannot be controlled for multiple vocational qualifications.

### Basic structure of the demand forecast for FTEs in nursing professions

**Figure 2:**
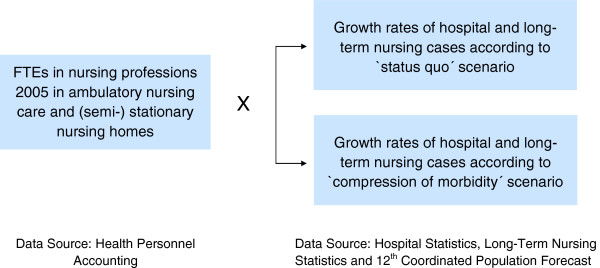
Components of the demand forecast; FTE, full-time equivalent.

The Federal Statistical Office composed and published two projections of the expected number of hospital cases and long-term care recipients to be treated by ambulatory nursing care or at (semi-) stationary nursing homes – the 'status quo scenario' and the 'compression of morbidity' scenario [1]. The status quo scenario assumes that the number of hospital cases and long-term care recipients depend solely on demographic developments. For the forecast of the number of hospital cases, present age and gender-specific hospital diagnosis probabilities (in five-year age groups) from years 2006, 2007 and 2008 were kept constant and applied to the predicted population, assuming a net migration inflow of 100 000 persons per annum from year 2014 onward [11]. The same approach was adopted for the forecast of the number of those needing long-term care. In this case, age and gender-specific rates of care from years 2005 and 2007 were extrapolated to the predicted population. The compression of morbidity scenario assumes that as life expectancy rises, people will remain healthy longer and will not be in need of care until a later stage of life [12]. Therefore, current age and gender-specific hospital diagnosis or care probabilities were shifted into higher age groups in line with the rise in life expectancy. An 'expansion of morbidity' [13] scenario has not been calculated because treatment rates in age cohorts in Germany declined by approximately 2% between 1999 and 2007 [1]. The basic structure of the demand projection for FTEs in nursing professions is shown in Figure 2. It assumes a constant ratio between the number of nursing professionals and the number of patients from year 2005 onwards. This only applies when the following marginal conditions remain constant at the same time:

•Average duration of patient hospital stay.

•Division of tasks between medical and non-medical staff.

•Proportion of persons cared for by family members only and proportion of persons receiving care through ambulatory nursing care or (semi-) stationary nursing homes.

•Distribution of those in need of care in the care levels I, II and III of the German system.

### Basic structure of the supply forecast for FTEs in nursing professions

**Figure 3:**
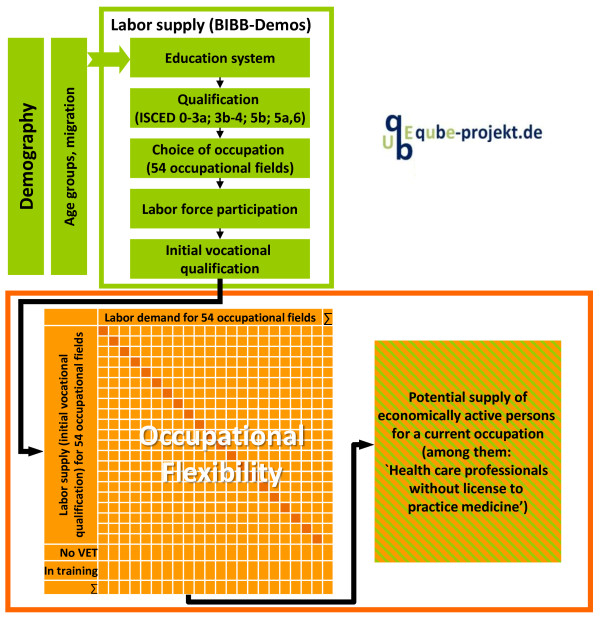
**Structure of the BIBB-DEMOS model in the BIBB-IAB Qualification and Occupational Field Projections.** Source: QuBe-project. BIBB, Federal Institute for Vocational Education and Training; VET, vocational education and training.

The labour supply projection was calculated using the BIBB-DEMOS stock-flow model [14,15] from the German BIBB-IAB Qualification and Occupational Field Projections (QuBe project) [15,16] (see Figure 3). The BIBB-DEMOS model uses information from the Microcensus regarding participation in education (ISCED: 1-3a; 3b-4; 5b; 5a and 6, 54 initial vocational qualifications [17]) and participation in employment. The absolute change and relative fluctuation in age cohorts, separated for women and men, were derived from the 12th Coordinated Population Projection, assuming a net migration inflow of 100 000 persons per annum starting from year 2014 (like the demand forecast) [11]. The labour supply was thus determined by taking several interconnected processes into account: demographic developments determine not only distribution in the individual age-cohorts and by gender, but also the size of the potential labour force, namely the number of people who are age 15 years or older. Additionally, the cohort-specific education trend typically exhibits rising levels of education, particularly among women, and also determines the duration of education. Labour force participation is also subject to change over time and was therefore estimated separately for each combination of age, gender and qualification. This model also takes into account the raising of the regular retirement age from 65 to 67 years.

Based on information regarding the initial vocational qualification of the labour force in 2005, occupational flexibility matrices were calculated to identify (1) the share of people who work in the occupational field in which they originally trained – stayers – and (2) the share of people who migrate from their initial vocational qualification to another occupational field – movers. Using these occupational flexibility matrices, the QuBe project calculated the potential supply of labour for each occupational field. The respective level of occupational flexibility was assumed to remain stable over the entire forecasting period [18]. Future labour force participants were assigned to one of the 54 occupational fields based on their education-specific (initial vocational qualification), skill-specific (highest level of formal qualification) and age-specific occupational flexibility (Figure 3).

To forecast the potential supply of nursing professionals, all employed and unemployed persons age 15 years or older who obtained their highest vocational qualification in occupational field 48 (health care professionals without license to practice medicine) were projected up to the year 2025 using the BIBB-DEMOS model [14]. Occupational flexibility was determined on the basis of the 2005 Microcensus for occupational field 48 and also applied to the nursing professions. It was also assumed that occupational flexibility would remain the same over the entire forecast period. The Microcensus and the Health Personnel Accounting system were then used to first calculate the share of workers in nursing professions in occupational field 48 and then to identify the number of FTEs in nursing professions. The number of FTEs in nursing professions was then adjusted to reflect the scale used in the Health Personnel Accounting system.

**Figure 4:**
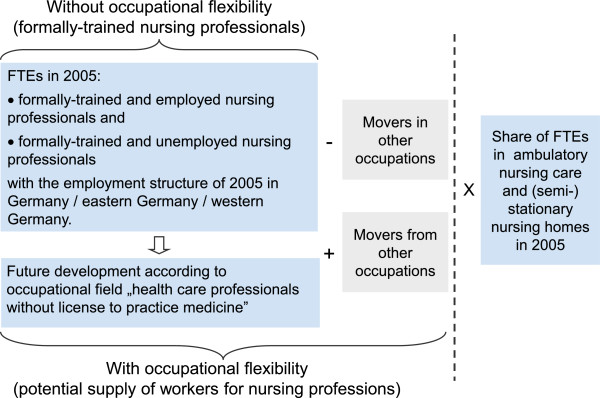
**Components of the supply forecast.** FTE, full-time equivalent.

The proportion of FTEs in nursing professions at health care providers in 2005 was calculated on the basis of data from the Health Personnel Accounting system and was held constant during the forecast period. In terms of type of employment (full-time, part-time and marginal employment), and number of weekly hours worked, the same structures were assumed for the following groups: formally trained nursing professionals and workers in nursing professions; employed and unemployed formally trained nursing professionals; workers in nursing professions at health care providers and all workers in nursing professions. Figure 4 provides an overview of the components of the supply forecast.

## Results

### Effects of occupational mobility

**Figure 5:**
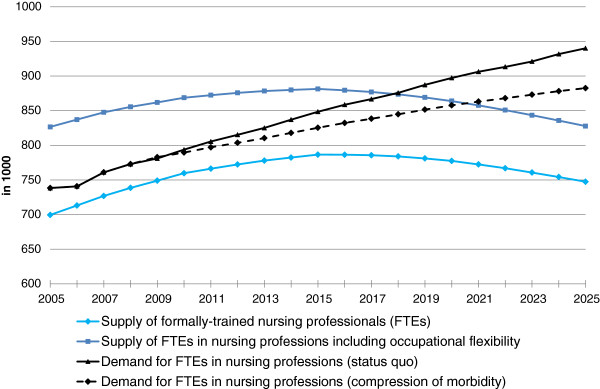
**Demand and supply forecast for full-time equivalents (FTEs) in nursing professions, with and without occupational flexibility - employment structure for Germany as a whole.** Source: Federal Institute for Vocational Education and Training: BIBB-DEMOS model. Federal Statistical Office: German Microcensus, Health Personnel Accounts, forecast of hospital cases and persons needing care; own calculations.

The black lines in Figure 5 show the demand for FTEs in nursing professions. According to the status quo scenario, the demand for FTEs in nursing professions at health care providers will rise by a total of 27.3% between the year 2005 (the start of the projection) and 2025. According to the compression of morbidity scenario, the demand for FTEs in nursing professions will increase by a total of 19.5% by the year 2025.

In the BIBB-DEMOS model, the age-, gender- and qualification-specific characteristics (including the initial vocational qualification) are extrapolated by age cohort. The supply of formally trained nursing professionals that is shown by the light blue line in Figure 5 is therefore the supply of FTEs with at least one year of training in a nursing profession, including anticipated new entrants into the labour market (graduates in nursing professions) and leavers (for example, due to retirement) from the labour market. The fact that some nurses will exit the profession in which they have been trained [9], and that semi-skilled labour nurses may enter the occupational field, is not accounted for. Therefore, the dark blue line in Figure 5 indicates the potential labour supply accounting for occupational flexibility. The supply figure in 2005 is then significantly larger than the demand for FTEs in nursing professions and reflects a rather realistic picture of the year 2005. However, when the employment structure for all nursing professionals resembles the employment structure in Germany in 2005 in the future, a shortage of nursing professionals will nonetheless emerge starting 2018 according to the status quo scenario and starting 2021 according to the compression of morbidity scenario, even when the inflow of semi-skilled persons is taken into account. Demographic and qualification developments exhibit the strongest effects, as demonstrated by the difference between the dark and light blue line in Figure 5, which shrinks by about a third between 2005 and 2025. This is due to constant occupational flexibility rates from 2005 to 2025 and a decline in the unskilled labour force that is moving primarily into auxiliary (geriatric) nursing occupations. Consequently, in order to identify further labour potential it is necessary to examine occupational mobility to and from nursing professions.

### Occupational flexibility and areas of potential supply

In the following, we present an analysis of occupational flexibility in nursing professions and in occupational field 48 (health care professionals without license to practice medicine) that was conducted on the basis of the Microcensus 2005. We chose the year 2005 because it is the starting year for the projections. However, results for the years 2006 through 2008 differ only slightly from the results presented below. The following results examine two different levels: the level of initial vocational qualification shows the behaviour of those who have been trained for at least one year in a nursing profession (formally trained nursing professionals), whereas current occupation shows everyone who is employed in a nursing profession and in occupational field 48, disregarding their formal occupational qualification. Persons who remain in the occupation they have learned are designated as stayers. Persons whose current employment is different from their initial vocational qualification are called movers.

Table 2 shows that 75.4% of persons who have been formally trained for at least one year in occupational field 48 also work in this occupational field. The occupational flexibility of formally trained nursing professionals differs somewhat from the flexibility of formally trained workers from the superordinate occupational field 48. The proportion of stayers amongst formally trained nursing professionals is 9.1 percentage points higher (74.8% as compared to 65.9%) than amongst all skilled workers in occupational field 48. This means that on average, formally trained nursing professionals exit their initial occupation less frequently than formally trained workers in occupational field 48. Additionally, it is evident that nursing professions appear to benefit somewhat more from occupational inflow than the entire occupational field 48 because the distribution between formally trained (stayers) and semi-skilled nursing professionals (movers into nursing professions) does not materially differ from the qualification structure (stayer-mover ratio) of workers in occupational field 48 (level: current occupation in Table 2).

**Table 2:** Occupational mobility in nursing professions

**Three-digit level of occupational classification: initial vocational qualification**		**Mover out of 3-digit occ.**	**Stayer in 3-digit occ.**	**Mover out of OF 48**	**Stayer in OF 48**	**Number of cases**
853	Formally trained^a^ nurses/midwives	23.6%	76.4%	14.2%	85.8%	6182
854	Formally trained auxiliary nurses	50.1%	49.9%	30.3%	69.7%	363
864	Formally trained geriatric nurses	25.6%	74.4%	12.7%	87.3%	1623
-	All formally trained nursing professionals	25.2%	74.8%	14.7%	85.3%	8168
OF 48: health care professionals without license to practice medicine		34.1%	65.9%	24.6%	75.4%	16 999
**3-digit level of occupational classification: current occupation**		Mover into 3-digit occ.	Stayer in 3-digit occ.	Mover into OF 48	Stayer in OF 48	Number of cases
853	Nurses/midwives	24.9%	75.1%	19.4%	80.6%	6255
854	Auxiliary nurses	85.3%	14.7%	70.6%	29.4%	1274
864	Geriatric nurses	62.8%	37.2%	52.5%	47.5%	3222
-	All nursing professions	43.6%	56.4%	35.5%	64.5%	10 751
OF 48: health care professionals without license to practice medicine		42.9%	57.1%	34.6%	65.4%	19 577

Occ., occupation; OF, occupational field; ^a^formally trained: at least one year of training.

Source: Federal Statistical Office: Microcensus 2005; own calculations.

Unfortunately, the Microcensus is not suitable for analyzing why formally trained nursing professionals exit the occupation that they learned, to work in another area. At this stage, we can only point to a few factors, such as exhaustion or working conditions that have been identified in other studies [9,19]. Also, we cannot tell why persons with a different occupational background chose to work as semi-skilled workers in a nursing profession. However, by comparing the number of stayers at the initial vocational qualification and at the current occupation levels we were able to quantify occupational inflow to and outflow from the nursing professions.

Table 3 shows that formally trained nurses are more likely to hold a full-time or part-time contract. Also, the proportion of stayers and movers are similarly distributed for the two different types of contracts. Thus, it can be concluded that converting part-time into full-time positions (or vice versa) is unlikely to have a strong effect on the likelihood for a formally trained nursing professional to move out of the nursing field. Only formally trained nursing professionals with a marginal employment contract seem to be more likely to work in an occupation outside the one in which they originally trained. This indicates that marginal employment contracts have a negative impact on the supply of nursing professionals in two different ways: marginal employment is not only reducing the overall amount of hours worked in the nursing field; it also diminishes the share of formally trained nursing professionals in the nursing field.

**Table 3:** Occupational mobility by hours of work

**Three-digit level of initial vocational qualification**		**Full-time**			**Part-time**			**Marginal employment**		
		**Mover out of OF 48**	**Stayer in OF 48**	**Number of cases**	**Mover out of OF 48**	**Stayer in OF 48**	**Number of cases**	**Mover out of OF 48**	**Stayer in OF 48**	**Number of cases**
853	Formally trained^a^ nurses/midwives	11.8%	88.2%	3723	12.3%	87.7%	1946	39.3%	60.7%	513
854	Formally trained auxiliary nurses	29.1%	70.9%	250	NP	NP	NP	NP	NP	NP
864	Formally trained geriatric nurses	10.6%	89.4%	1011	11.7%	88.3%	496	36,4%	63,6%	116
-	All formally trained nursing professionals	12.4%	87.6%	4984	12.7%	87.3%	2523	39.3%	60.7%	661
OF 48: health care professionals without license to practice medicine		22.1%	77.9%	9865	22.5%	77.5%	5177	42.6%	57.4%	1895

OF, occupational field; ^a^formally trained: at least one year of training; NP, not published due to small sample size.

Source: Federal Statistical Office: Microcensus 2005; own calculations.

Research on the intent to leave the nursing profession has shown that in Germany it is the younger and older age cohorts in particular that consider leaving this profession [9]. These findings cannot be verified on the basis of Microcensus data because the percentage of stayers can be observed to decline with age. While the proportion of stayers is 90.5% for the cohort age 15 to 34 years, it is at its lowest, at 80.4%, for the 50 years and above age group (see Table 4). This shows that young nurses and midwives in particular have a low level of mobility. Of 15- to 34-year-old nurses and midwives, 92.5% are classified as stayers, which is a substantial divergence from the proportion of stayers in the other age groups. In contrast, formally trained geriatric nurses exhibit approximately the same stayer-rate in all age cohorts.

**Table 4:** Occupational mobility by age cohort

**Three-digit level of initial vocational qualification**		**Age 15 to 34 yrs**			**Age 35 to 49 yrs**			**Age 50 yrs and over**		
		**Mover out of OF 48**	**Stayer in OF 48**	**Number of cases**	**Mover out of OF 48**	**Stayer in OF 48**	**Number of cases**	**Mover out of OF 48**	**Stayer in OF 48**	**Number of cases**
853	Formally trained^a^ nurses/midwives	7.5%	92.5%	1658	15.3%	84.7%	3214	20.9%	79.1%	1310
854	Formally trained auxiliary nurses	29.5%	70.5%	144	33.8%	66.2%	147	24.1%	75.9%	72
864	Formally trained geriatric nurses	10.8%	89.2%	444	13.3%	86.7%	831	13.8%	86.2%	348
-	All formally trained nursing professionals	9.5%	90.5%	2246	15.5%	84.5%	4192	19.6%	80.4%	1730
OF 48: health care professionals without license to practice medicine		18.1%	81.9%	5370	26.9%	73.1%	8434	30.0%	70.0%	3163

OF, occupational field; ^a^formally trained: at least one year of training.

Source: Federal Statistical Office: Microcensus 2005; own calculations.

### Effects of full-time and part-time employment

**Figure 6:**
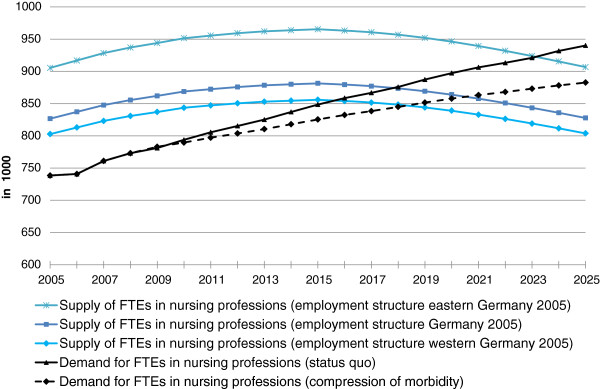
**Demand and supply forecast for full-time equivalents (FTEs) in nursing professions, with occupational flexibility and different employment structures.** Source: Federal Institute for Vocational Education and Training: BIBB-DEMOS model. Federal Statistical Office: German Microcensus, Health Personnel Accounts, forecast of hospital cases and persons needing care; own calculations.

Microcensus data show that employment structures in eastern and western Germany differ significantly. We calculated three different scenarios for FTEs in nursing professions based on the employment structure in East and West Germany and Germany as a whole, accounting for occupational flexibility of the workforce. Figure 6 shows that in contrast to an employment structure of Germany as a whole, a theoretical shortage of nursing professionals would occur two years earlier if a West German employment structure was to prevail. In the case of an employment structure like in eastern Germany, supply of FTEs in nursing professions would only be sufficient by the year 2025 (surplus of 24 000 FTEs in nursing professions) when demand develops as foreseen in the compression of morbidity scenario and the employment structure for all nursing professionals in the future resembled the East German structure in 2005. This shows that Germany could increase its supply of FTEs in nursing professions by approximately 9.5% if everyone was to work the same number of hours as in East Germany. These insights induced us to investigate the motivation behind full-time and part-time work.

### Full-time and part-time employment in nursing professions

**Figure 7:**
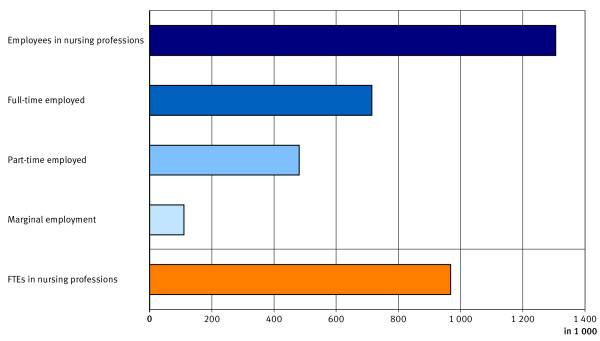
**Employed nursing professionals, by employment structure 2005.** Source: Federal Statistical Office: Health Personnel Accounts 2010; FTE full-time equivalent.

In 2005, around 1.3 million workers were employed in nursing professions (see Figure 7). Many nursing professionals worked on a part-time basis or were marginally employed (45.3%). Therefore, the number of FTEs in nursing professions – 968 000 – was well below the actual number of workers. In 2005, an average of 1.3 employees held one full-time position in the nursing professions.

The primary reason for the large proportion of part-time and marginally employed workers is the large share of women working in nursing professions, representing 84% of all nursing professionals (see Table 5). While 21.1% of men employed in nursing professions worked on a part-time basis or were marginally employed, this figure was nearly one half (49.9%) among women. Additionally, the 2005 Microcensus shows that 29.8% more women were in full-time employment in eastern compared to western Germany. Furthermore, women in eastern Germany who were employed on a part-time basis or were marginally employed, worked on average 4.9 and 4.7 hours more per week respectively, than their counterparts in western Germany. This might be due to the greater availability of child care services in East Germany. Here the share of children under the age of three in childcare facilities is almost four times larger than in the western states (38% in eastern Germany, 10% in western Germany) [20].

**Table 5:** Nursing professionals by gender and type of occupation 2005

**Three-digit level of occupational classification: current occupation**		**Employed persons (1000's)**	**Employed persons who were women (%)**
853	Nurses/midwives	763	86.4
854	Auxiliary nurses	232	71.1
864	Geriatric nurses	311	87.4
	All nursing professions	1306	83.9

Source: Federal Statistical Office: Health Personnel Accounts 2010; own calculations.

Female nursing professionals in eastern Germany also state significantly different reasons for working on a part-time basis than in western Germany (see Table 6). In eastern Germany, the main reason for part-time employment was that no full-time positions were available (46.2%); in western Germany it was personal or due to family commitments (69.0%).

**Table 6:** Main reasons for part-time/marginal employment of female nursing professionals in eastern and western Germany in 2005

**Main reasons**	**Eastern Germany, %**	**Western Germany, %**
Illness and accidents	NP	2.8
Personal or family commitments	31.0	69.0
School or other education	NP	2.9
No full-time job available	46.2	11.5
Full-time employment not sought due to other reasons	15.3	12.5
Not stated	NP	1.3
Sum	100	100

NP, not published due to small sample size.

Source: Federal Statistical Office: Microcensus 2005; own calculations.

## Conclusions

Our forecast is not intended for use in detailed human resource planning [21]. This would require a model with a more complex structure that takes into account nursing graduates and mobility at regional level, since training for nursing professions falls under the jurisdiction of Germany's 16 *Bundesländer* (federal states). However, unlike most models developed for human resource planning in the health sector, our BIBB-DEMOS supply model also takes into account the supply of labour for other occupations. This allowed us to include changes of occupation to and from nursing professions in the supply forecast. Due to the already increasing demand of nursing professions the number of persons employed as auxiliary (geriatric) nurses has strongly increased in recent years [8]. In contrast to graduate (geriatric) nurses, it is also possible for semi-skilled labour nurses with less than one year of training to work in this occupation [2]. This potential inflow of labour has to be considered in a supply forecast of nursing professions when the demand forecast also includes auxiliary (geriatric) nurses.

The results show that in the years up to 2025 it will no longer be possible to meet the growing demand for nursing professionals if current age- and gender-specific hospital diagnosis and care probabilities remain constant (status quo scenario) and the current structure of full-time and part-time employment remains the same (see Figure 6). Today it is already necessary to train workers from non-nursing fields to support formally trained nursing professionals as semi-skilled workers (see Figure 5).

Besides examining shortages, this paper also outlines possible solutions for meeting the challenge of the shortage of nursing professionals. Areas for possible action revolve around occupational flexibility and full-time and part-time employment in nursing professions. Furthermore, the supply forecast also assumes rising participation rates due to the adjustment of the statutory retirement age from 65 to 67 years and a net migration inflow of 100 000 persons starting in the year 2014. However, we forwent investigating the effects of participation rates and migration on the supply of nursing professionals in favour of examining the new concept of occupational flexibility. We hope that our research provides impetus for researchers in other countries to use the information provided by the LFS data (Table 1), to obtain more insights into the occupational mobility of nursing professionals and to enrich their national forecasts.

Our analysis of occupational flexibility in the nursing field shows that the number of persons working in the field of their initial vocational qualification is very large regardless of their age or type of work contract (that is, full-time or part-time). Young workers in the nursing field are most loyal to the occupation in which they trained compared to the other age groups. Thus, increasing recruitment of young people to train as nurses can moderate the shortage of the future supply of nurses. Additionally, the large proportion of semi-skilled workers amongst auxiliary (geriatric) nurses also shows that there is worker inflow to these occupations. Despite this, in future the attractiveness of the nursing professions has to increase due to the fact that the growing shortage of workers implies an increasing scope in their choice of occupation. Hence, remuneration and other aspects such as improved working environment [22,23] and type of employment contract will play a role. Therefore, simply using employment contracts for a small number of hours per month (marginal employment) to increase the number of nursing professionals will not counteract the shortage of formally trained nursing professionals, because they are more likely to stay in the field if they have a full-time or part-time contract (see Table 3). Nevertheless, a retrospective examination of employment contracts starting from the year 2000 did not reveal any discernible trend towards a growing number of full-time employment contracts. Instead, part-time and marginal employment contracts are becoming increasingly important.

One main reason, particularly in western Germany, for part-time employment among female nursing professionals is personal or family commitment (see Table 6). Thus, some workers would always decline if offered an increased number of working hours. One reason for this could be the difficulty for some female nursing professionals of reconciling work with raising a family. Therefore, more family-friendly approaches to the work-life balance in nursing professions and an expansion of child care facilities for children under the age of 3 years, especially in western Germany, may help to increase the supply of nursing professionals towards the eastern German employment structure scenario (Figure 6).

## Endnotes

### Occupations in the occupational field 48: health care professionals without license to practice medicine

The supply of nursing professionals in this paper was derived from the supply forecast for the occupational field, health care professionals without license to practice medicine, which was calculated by the BIBB-DEMOS model. This occupational field comprises the following occupational categories from the KldB 92: allied health professions (category 805); non-medical practitioner (category 851); physiotherapist, certified masseurs, medically qualified lifeguard (category 852); nurses and midwives (category 853); auxiliary nurses (category 854); assistant dieticians and nutrition specialists (category 855); doctor's assistants, dental assistants and veterinary employees (category 856); medical laboratory assistants and related occupations (category 857); pharmaceutical-technical assistants (category 858); therapeutic professions otherwise not stated (category 859); geriatric nurses and auxiliary geriatric nurses (category 864); remedial therapists (category 866).

### The German Microcensus

The German Microcensus (German LFS), is a representative, annually rotating household survey of approximately 370 000 German households, covering approximately 1% of the German population (some 830 000 persons). One fourth of the sample is replaced every year. Information is collected mainly in face-to-face interviews (77%); households that cannot be contacted by the interviewer are sent a questionnaire by post. It is permissible for one person (18 years or older) to give answers for other household members. These proxy interviews account for approximately one fourth of the interviews [24]. Due to the large sample size (the response rate is generally about 97%), the results are regarded as highly reliable and are used for the official statistics on the economic and social situation of the population. For the years since the survey year 2005, it is possible to reconstruct the workforce's initial vocational qualification by matching workers' specializations with the appropriate occupational classifications and formal qualification levels [10]. However, as the major field of study is only surveyed with the highest qualification achieved, it cannot be controlled for multiple vocational qualifications.

### Qualification for nursing professions

Graduate geriatric nurses specialize in nursing elderly persons and work primarily in geriatric departments in hospitals or homes for the elderly. Graduated nurses are usually employed in hospitals. Both professions require at least three years of training. As a rule, graduate geriatric nurses must complete a full training programme in order to become a graduate nurse. The same applies to graduate nurses wishing to be a graduate geriatric nurse. However, since the curricula are similar, individuals can receive credit for content covered during their previous training and the duration of the additional training programme can consequently be shortened to approximately one year. However, in practice it is possible to employ geriatric nurses as regular nurses and vice versa but this always depends on the specific duties that have to be performed (for example, without additional training, geriatric nurses are not allowed to give intravenous injections). Since the training content for graduate nurses is broader, it is somewhat easier for graduate nurses to work as geriatric nurses than vice versa. Auxiliary nurses (training period of one or two years) are also able to work as auxiliary geriatric nurses (one-year training period) but not the other way around. In addition to the option of undergoing an additional training programme for auxiliary (geriatric) nurses, it is also possible to carry out some nursing duties as (geriatric) nursing assistants following completion of a few weeks of training. These (geriatric) nursing assistants are classified as auxiliary nurses or geriatric nurses in the KldB 92; see also [2].

## Abbreviations

BIBB: Federal Institute for Vocational Education and Training (*Bundesinstitut für Berufsbildung*); FTE: full-time equivalent; IAB: Institute for Employment Research (*Institut für Arbeitsmarkt und Berufsforschung*); ISCED: International Standard Classification of Education; ISCO: International standard classification of occupations; KldB 92: German classification of occupations 1992 (*Klassifikation der Berufe 1992*); LFS: (European) Labour Force Survey; OF: Occupational field; VET: Vocational education and training.

## Competing interests

The authors declare that they have no competing interests.

## Authors’ contributions

TM was responsible for the supply side and the calculation of occupational flexibility. AA was responsible for the demand side and the calculation of different employment structures on the supply side. Both authors contributed to writing the final manuscript and approve its content.

## Supplementary Material

Additional file 1 **Former publication of the study results in German: Afentakis A, Maier T: Projektionen des Personalbedarfs und –angebots in Pflegeberufen bis 2025.** Wirtschaft und Statistik 2010, **11**: 990-1002. Click here for file
