# Supplementary material for: Forecasting supply and demand in nursing professions: impacts of occupational flexibility and employment structure in Germany
Source: Hum Resour Health. 2013 Jun 5;11:24. doi: 10.1186/1478-4491-11-24 (PMC3693609; doi:10.1186/1478-4491-11-24)
Supplement: Additional file 1 — Former publication of the study results in German: Afentakis A, Maier T: Projektionen des Personalbedarfs und –angebots in Pflegeberufen bis 2025. Wirtschaft und Statistik 2010, 11: 990-1002. [file 1478-4491-11-24-S1.pdf]

Dipl.-Sozialwissenschaftlerin Anja Afentakis (Statistisches Bundesamt),
Dipl.-Verwaltungswissenschaftler Tobias Maier (Bundesinstitut für Berufsbildung)

Projektionen des Personalbedarfs und -angebots in Pflegeberufen bis 2025

Die Pflege kranker und älterer Menschen ist sehr personalintensiv und muss überwiegend von qualifizierten Personen geleistet werden. Eine schrumpfende und zugleich alternde Gesellschaft bringt hier neue Herausforderungen mit sich und lässt die bestehenden Pflegeeinrichtungen schnell an ihre Grenzen stoßen. Werden die Berufsabschlüsse des Pflegepersonals betrachtet, so wird deutlich, dass bereits heute fachfremdes Personal in der Pflege eingesetzt wird, um den Bedarf decken zu können. Die Bedarfs- und Angebotsprojektionen zeigen, dass ein Pflegepersonalmangel in Zukunft voraussichtlich auch nicht durch die zusätzliche Beschäftigung ungelernter beziehungsweise angelernter Pflegekräfte verhindert werden kann. Zu welchem Zeitpunkt ein Pflegepersonalmangel auftreten wird, hängt neben der künftigen Zahl an ausgebildeten und fachfremden Pflegekräften auch von der Beschäftigungsstruktur (Anteil der Vollzeit-, Teilzeit- und geringfügig Beschäftigten sowie der geleisteten Wochenarbeitszeit) in Pflegeberufen ab. Daher wurden bei den Angebotsprojektionen unterschiedliche Beschäftigungsmodelle berücksichtigt.

In diesem Aufsatz wird zunächst die Bedarfs- und Angebotsentwicklung für den gesamten deutschen Arbeitsmarkt bis zum Jahr 2025 dargestellt. Zudem erfolgt eine Analyse nach Qualifikationsstufen sowie eine Bedarfs- und Angebotsprojektion für die Gesundheitsberufe ohne Approbation, zu denen auch die Pflegeberufe gehören. Anschließend werden Bedarfs- und Angebotsprojektionen explizit für Pflegeberufe erstellt. Während der künftige Erwerbstätigenbedarf für die Gesamtwirtschaft und die Gesundheitsberufe ohne Approbation aus der bisherigen Entwicklung der Zahl der Erwerbstätigen projiziert wurde, wird der Bedarf in Pflegeberufen nachfrageorientiert hergeleitet aus der zu erwartenden Zahl der Krankenhausfälle und der Pflegebedürftigen. Die Angebotsentwicklung für die Gesundheitsberufe ohne Approbation wird auf die Pflegeberufe übertragen. Abschließend werden die Angebotspotenziale und die berufliche Flexibilität der Pflegeberufe analysiert.

1 Arbeitsmarkt und bildungssystembezogene Betrachtung

Der deutsche Arbeitsmarkt steht vor tiefgreifenden Veränderungen. Bei einem weiterhin hohen Bedarf an *Erwerbstätigen* wird das Angebot an *Erwerbspersonen*[1]) in den kommenden Jahren deutlich sinken und den Bedarf nicht mehr decken können. Dies zeigen die Qualifikations- und Berufsfeldprojektionen, die vom Bundesinstitut für Berufsbildung (BIBB) und vom Institut für Arbeitsmarkt- und Berufsforschung der Bundesagentur für Arbeit (IAB)[2]) in Zusammenarbeit mit dem Fraunhofer-Institut für Angewandte Informationstechnik FIT und der Gesellschaft für Wirtschaftliche Strukturforschung mbH erstellt wurden.[3]) Weiter wird gezeigt, dass Zeitpunkt und Ausmaß des Arbeitskräftemangels von

1) Mit Erwerbstätigen sind im Folgenden alle Personen in ihrer Haupterwerbstätigkeit gemeint. Erwerbspersonen sind alle dem Arbeitsmarkt zur Verfügung stehenden Personen ab 15 Jahren. Sie setzen sich aus Erwerbstätigen und Erwerbslosen zusammen. Bei der Ermittlung des Angebots bleiben Nicht-Erwerbspersonen unberücksichtigt. Dies sind alle Personen im Alter von 15 Jahren und älter, die bewusst keiner Erwerbstätigkeit nachgehen möchten.

2) Helmrich, R./Zika, G. (Hrsg.): „Beruf und Qualifikation in der Zukunft. BIBB-IAB-Modellrechnungen zu den Entwicklungen in Berufsfeldern und Qualifikationen bis 2025.“, Schriftenreihe „Berichte zur beruflichen Bildung“, Bonn 2010.

3) Weitere Informationen unter *www.qube-projekt.de*.

der Qualifikationsstufe abhängig sind. Auch in den Gesundheitsberufen ohne Approbation kann der Erwerbstätigenbedarf in absehbarer Zeit nicht mehr durch das Erwerbspersonenangebot gedeckt werden.

1.1 Gesamtwirtschaftliche und qualifikationsabhängige Bedarfs- und Angebotsentwicklung

In Schaubild 1 werden der Erwerbstätigenbedarf und das Erwerbspersonenangebot für die Gesamtwirtschaft projiziert: Je nach Angebotsmodell geht die Zahl der Erwerbspersonen unterschiedlich stark zurück. So würde nach dem FIT-Modell[4]) bereits nach 2023 rein rechnerisch eine Vollbeschäftigung eintreten. Nach dem BIBB-DEMOS-Modell[4]) unterschreitet das Angebot an Erwerbspersonen erst zu einem späteren Zeitpunkt den Bedarf. Dies liegt vor allem daran, dass das BIBB-DEMOS-Modell höhere Erwerbsquoten als die FIT-Projektionen unterstellt. Hieraus kann generell abgeleitet werden, dass sich bei einem moderat steigenden Bedarf an Erwerbstätigen ab 2023 zunehmend ein flächendeckender Fachkräftemangel einstellen wird. Diese Entwicklung ist demografisch bedingt und wird sich bei höherer Zuwanderung zeitlich verzögern, aber nicht aufhalten lassen. Der in dieser Projektion unterstellte Zuwanderungssaldo von 100 000 Personen je Jahr liegt am oberen Rand der Zuwanderungssalden der letzten Jahre. Derzeit ist die Abwanderung aus Deutschland jedoch höher als die Zuwanderung. Setzt sich diese Entwicklung fort, würde sich das Angebot noch schneller verringern als in den Projektionsrechnungen angenommen. Der Mangel an Fachkräften wird jedoch in einzelnen Qualifikationsstufen schon früher sichtbar.

Das Angebot an Personen mit einer abgeschlossenen Berufsausbildung wird demografisch bedingt sinken und je nach Angebotsprojektion schon vor 2020, spätestens jedoch 2025 den entsprechenden Bedarf rein rechnerisch nicht mehr decken können. Schon vor diesem Zeitpunkt wird man bei diesem Qualifikationsniveau mit einem zunehmenden Fachkräftemangel konfrontiert sein, und damit werden neben den beruflichen Qualifikationen auch die Arbeitsbedingungen ein zentrales Entscheidungsmerkmal für oder gegen die Wahl eines bestimmten Berufes sein.

Der Bedarf an Arbeitskräften ohne eine abgeschlossene Berufsausbildung wird auf seinem aktuellen Niveau verbleiben beziehungsweise weiterhin leicht sinken. Das entsprechende Angebot wird seinerseits nur langsam zurückgehen, womit sich das bestehende Überangebot an Personen ohne Berufsabschluss nicht verringern wird. Diese Personengruppe wird auch in Zukunft keine besseren Beschäftigungschancen auf dem Arbeitsmarkt vorfinden. Um dem Fachkräftemangel auf mittlerem Qualifikationsniveau zu begegnen, müssen größere Anstrengungen unternommen werden, dieser Personengruppe eine qualifizierte Ausbildung zu vermitteln.

Schaubild 1

Entwicklung der Erwerbstätigen und Erwerbspersonen

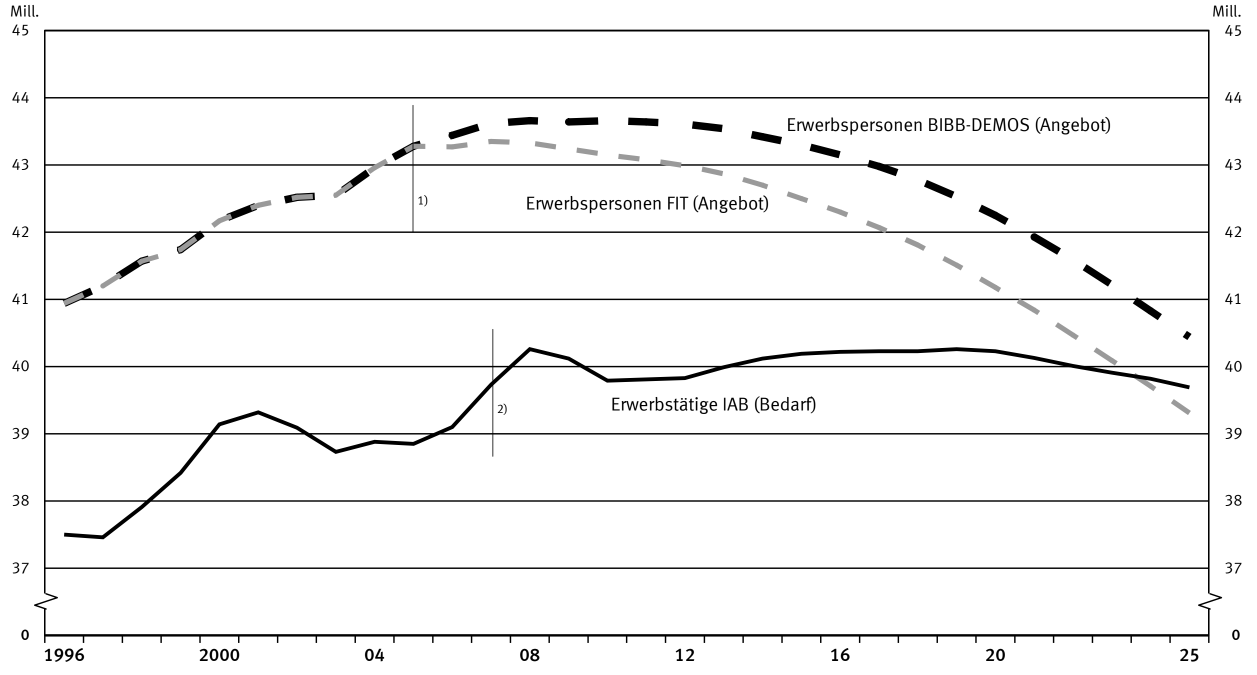

1) Ab 2005 Projektionsergebnisse. – 2) Ab 2007 Projektionsergebnisse.

Quelle: Helmrich, R./Zika, G., Fußnote 2 im Text, hier: S. 19.

2010 - 01 - 0812

4) Nähere Informationen zu den verwendeten Modellen enthält der folgende Methodenkasten.

BIBB-IAB-Qualifikations- und Berufsfeldprojektionen

Die BIBB-IAB-Qualifikations- und Berufshauptfeldprojektionen [Helmrich, R./Zika, G. (Fußnote 2) 2010; *www.qube-projekt.de*] sind koordinierte Angebots- und Bedarfsprojektionen auf der Grundlage einer gemeinsamen Datenbasis und einheitlich definierter Berufsfelder.1) Hierbei handelt es sich um 54 BIBB-Berufsfelder, die zusammengefasst auf der Ebene der Berufsordnungen [Dreisteller der Klassifikation der Berufe 1992 (KldB 92)] jeweils gleiche Tätigkeitsmerkmale und Branchendominanz aufweisen.

Datenbasis:

Auf der Grundlage der Mikrozensen mehrerer Jahre sind sowohl der Bestand an Erwerbstätigen nach ausgeübtem Beruf und Branchenzugehörigkeit als auch die Erwerbspersonen nach Qualifikationsniveau, Alter und Geschlecht aufbereitet worden. Für das Erhebungsjahr 2005 steht der erlernte Beruf der Erwerbspersonen zur Verfügung. Der Mikrozensus weist gegenüber den Volkswirtschaftlichen Gesamtrechnungen tendenziell eine geringere Zahl an Erwerbstätigen aus. Daher wurden die Erwerbstätigenzahlen des Mikrozensus mit einem iterativen Randsummenanpassungsverfahren auf die Erwerbstätigenzahlen der Volkswirtschaftlichen Gesamtrechnungen hochgerechnet. Die Verteilung der Variablen „Alter“, „Geschlecht“, „Bildungsniveau“ und „erlernter Beruf“ unterteilt nach 59 Wirtschaftszweigen wurde nicht verändert.

Bedarfsprojektion:

Bei der Bedarfsprojektion werden die Erwerbstätigen nach ihrem ausgeübten Beruf in die 54 BIBB-Berufsfelder unterteilt. Innerhalb dieser Berufsfelder wird nach vier Qualifikationsniveaus unterschieden, indem der höchste berufliche Abschluss der Erwerbstätigen berücksichtigt wird. Die Bedarfsprojektion stellt für die Jahre von 1996 bis 2007 (Ist-Zahlen) bzw. bis 2025 (Projektion) den realisierten Bedarf (Erwerbstätige) dar. Die Fortschreibung der Erwerbstätigen wird mit dem IAB-INFORGE-Modell vorgenommen. Dabei handelt es sich um ein makroökonometrisches Input-Output-Modell, das auf dem Konstruktionsprinzip „bottom-up“ nach 59 Wirtschaftszweigen, 59 Gütergruppen, 43 Verwendungsarten differenziert.

Angebotsprojektionen:

Die Projektion des Arbeitsangebots wurde mit dem Ziel der Modellpluralität mit zwei unabhängigen Modellen gerechnet, die auf der gleichen Systematik und Datenbasis wie die Bedarfsprojektion aufbauen. Dabei handelt es sich um das BIBB-DEMOS-Modell der Gesellschaft für Wirtschaftliche Strukturforschung mbH (GWS), das Schnittpunkte zum IAB-INFORGE-Modell aufweist, und um das etablierte Modell des Fraunhofer-Instituts für Angewandte Informationstechnik FIT, das bereits für die bekannten Prognosen der Bund-Länder-Kommission für Bildungsplanung und Forschungsförderung verwendet worden ist.

Die Angebotsprojektionen schreiben die Entwicklung der Erwerbspersonen nach vier Qualifikationsniveaus und ihrem erlernten Beruf (höchster zertifizierter Abschluss) fort. Die Informationen zum erlernten Beruf wurden aus dem Mikrozensus 2005 gewonnen. Zur Bestimmung des erlernten Berufs innerhalb der beiden Angebotsmodelle spielten vor allem das Alter, das Geschlecht, das Qualifikationsniveau und die Erwerbsbeteiligung eine Rolle.

BIBB-DEMOS-Modell:

Die Angebotsprojektion des BIBB-DEMOS-Modells (GWS) ist das Ergebnis mehrerer, in sich verschränkter Prozesse: Die absolute Veränderung und relative Verschiebung der Altersjahrgänge, getrennt nach Frauen und Männern, gehen auf die Bevölkerungsvorausberechnung des Statistischen Bundesamtes (12. koordinierte Bevölkerungsvorausberechnung) zurück. Die Bevölkerungsentwicklung legt nicht nur die Verteilung auf Altersjahrgänge und Geschlecht fest, sondern bestimmt letztendlich die Anzahl der potenziellen Erwerbspersonen, nämlich die Anzahl der Personen, die älter als 14 Jahre, aber jünger als 66 Jahre (68 Jahre) sind.2) Daran schließt sich der Qualifizierungsprozess an, der sich durch eine zunehmende Qualifizierung, insbesondere der Frauen, auszeichnet und auch die Dauer bis zum Erreichen eines formalen Ausbildungsabschlusses bestimmt. Die Entscheidung für oder gegen eine Erwerbsbeteiligung ist nicht nur alters- und geschlechtsspezifisch, sondern hängt auch von der erreichten formalen Qualifikation ab. Die Erwerbsbeteiligung ist zudem zeitlichen Veränderungen unterworfen. Auf Basis dieser Information findet die Wahl des zu erlernenden Berufs statt. Sie ist schließlich das Ergebnis einer geschlechts-, qualifikations- und altersspezifischen Verteilung.

FIT-Modell:

Auch die Arbeitsangebotsprojektionen des FIT-Modells differenzieren nach vier Qualifikationsstufen und 54 Berufsfeldern. Sie basieren auf mehreren Übergangsmodellen, nach denen beginnend mit einer Ausgangspopulation künftige Erwerbspersonenbestände über Zu- und Abgänge modelliert werden. Der gewählte Ansatz greift somit auf die drei Grundelemente einer Bevölkerungsprojektion, eines Übergangsmodells des Bildungssystems zur Ermittlung des Neuangebots sowie einer Analyse der Erwerbsbeteiligung zur Ermittlung der Restbestände zurück.

Saldierung von Bedarf und Angebot:

Für eine Gegenüberstellung von Angebot und Bedarf werden die Erwerbspersonen mit ihrem erlernten Beruf mit den benötigten Erwerbstätigen im ausgeübten Beruf mittels einer Flexibilitätsmatrix zusammengeführt. Diese Flexibilitätsmatrix basiert auf dem Mikrozensus des Jahres 2005, das für die Projektionen als Ausgangsjahr dient. Da Erwerbslose keinen Beruf ausüben, wird unterstellt, dass die Erwerbslosen dieselben beruflichen Flexibilitäten aufweisen wie die Erwerbstätigen. Das heißt beispielsweise, der Anteil der erwerbstätigen kaufmännischen Angestellten in Pflegeberufen wird auf alle (auch erwerbslose) kaufmännischen Angestellten übertragen.

Weitere Informationen zu den Modellen finden sich unter *www.qube-projekt.de*.

---

1) Tiemann, M./Schade, H.-J./Helmrich, R./Hall, A./Braun, U./Bott, P.: „Berufsfeld-Definitionen des BIBB auf Basis der Klassifikation der Berufe 1992“, Wissenschaftliche Diskussionspapiere, Heft 105, Schriftenreihe des Bundesinstituts für Berufsbildung, Bonn 2008.

2) Das Modell unterstellt eine Adaption des neuen Regelrenteneintrittsalters.

Schaubild 2

Projektion der Erwerbstätigen und der Erwerbspersonen nach Qualifikationsstufen

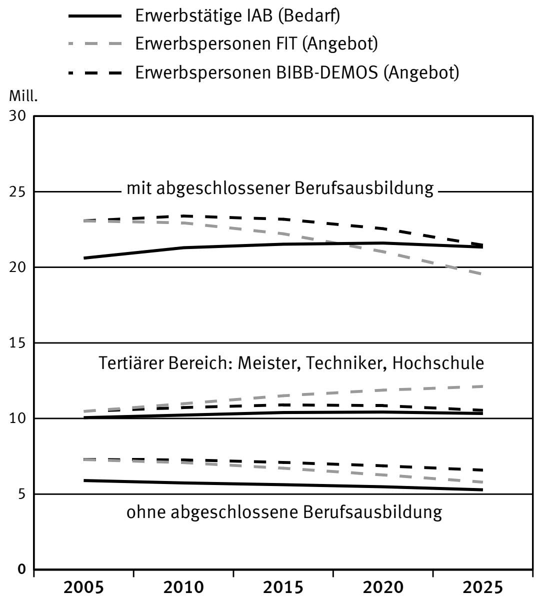

Quelle: Helmrich, R./Zika, G., Fußnote 2 im Text, hier: S. 13 bis 62.

2010 - 01 - 0813

Im tertiären Bereich werden sowohl Angebot als auch Bedarf an Erwerbstätigen weiter ansteigen – beide liegen bereits heute dicht beieinander. Bei Hochschulabsolventen rührt der zu erwartende Bedarf zu etwa gleichen Teilen aus dem Ersatzbedarf und dem durch den wirtschaftlichen Strukturwandel bedingten Neubedarf her. Der Ersatzbedarf wird ab 2020 aufgrund des Ausscheidens der geburtenstarken Jahrgänge (Baby-Boomer-Generation) deutlich anwachsen[5]). Das stärkere Überangebot an Erwerbstätigen mit Hochschulabschluss der FIT-Projektion im Gegensatz zum BIBB-DEMOS-Modell beruht auf der gegenwärtigen Zunahme der Zahl akademischer Abschlüsse.[6])

1.2 Abgrenzung der Gesundheitsberufe ohne Approbation

Die Gesundheitsberufe ohne Approbation sind größtenteils Berufe im tertiären Bereich. Sie sind im Berufsfeld 48 der 54 Berufsfelder umfassenden BIBB-Klassifikation definiert. Die hier im Fokus stehenden Pflegeberufe sind Teil des Berufsfeldes 48[7]). Im folgenden Abschnitt wird zunächst eine Angebots- und Bedarfsprojektion für das gesamte Berufsfeld 48 dargestellt. Hierauf aufbauend wird im Kapitel 2 die Angebots- und Bedarfsentwicklung in den Pflegeberufen prognostiziert.

1.3 Bedarfs- und Angebotsprojektion für das Berufsfeld 48

Im Jahr 2005 lag die Zahl der Erwerbstätigen im Berufsfeld 48 bei rund 2,4 Millionen. Jedoch hatten nicht alle Erwerbstätigen aus dem Berufsfeld 48 auch einen entsprechenden Beruf erlernt: Jede oder jeder Dritte hatte den höchsten beruflichen Abschluss in einem anderen Berufsfeld erworben oder arbeitete als un- oder angelernte Kraft im Berufsfeld 48 (siehe Tabelle 5). Geht man von der bisherigen Entwicklung (1996 bis 2007) des Arbeitskräftebedarfs aus, also der Entwicklung der Zahl der Erwerbstätigen im Berufsfeld 48 insgesamt, so wird dieser von 2005 bis 2025 um rund eine halbe Million Personen oder + 23,2 % ansteigen. Dieser Entwicklung werden Angebotsprojektionen gegenübergestellt, wobei einerseits der berufliche Abschluss im Berufsfeld 48 und andererseits die berufliche Flexibilität für einen Wechsel aus dem und in das Berufsfeld 48 berücksichtigt wird.

In Schaubild 3 wird bei der Angebotsprojektion der Erwerbspersonen keine berufliche Flexibilität berücksichtigt. Das heißt es wird unterstellt, dass alle erwerbstätigen und erwerbslosen Personen mit ihrem höchsten beruflichen Abschluss im Berufsfeld 48 auch tatsächlich in diesem Berufsfeld arbeiten bzw. arbeiten würden. Es wird deutlich, dass bereits im Jahr 2005 im betrachteten Berufsfeld 125 000 Erwerbstätige gefehlt hätten. Bis zum Jahr 2025 würde sich dieser Mangel auf 470 000 Erwerbstätige (Ange-

Schaubild 3

Projektion der Erwerbstätigen und Erwerbspersonen im Berufsfeld 48[1)] ohne berufliche Flexibilität

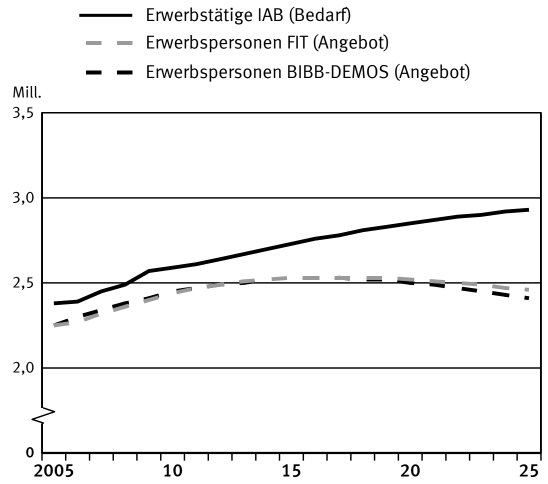

1) Gesundheitsberufe ohne Approbation.
Datenquelle: BIBB-IAB-Qualifikations- und Berufsfeldprojektionen, www.qube-projekt.de

2010 - 01 - 0814

5) Siehe Kalinowski, M./Quinke, H.: „Projektion des Arbeitskräfteangebots bis 2025 nach Qualifikationsstufen und Berufsfeldern“ in Helmrich, R./Zika, G. (Fußnote2), hier: Seite 103 ff.

6) Sekretariat der Ständigen Konferenz der Kultusminister der Länder in der Bundesrepublik Deutschland: „Vorausberechnungen der Studienanfängerzahlen 2009 – 2020 – Zwischenstand“ (*www.kmk.org/fileadmin/pdf/Statistik/Zwischenstand_Vorausberechnung_Studienanfaengerzahlen_2009_2020.pdf*; abgerufen am 4. November 2010).

7) Hierzu gehören die folgenden Berufsordnungen (BO) der Klassifikation der Berufe 1992: Gesundheitssichernde Berufe (BO 805), Heilpraktikerin und Heilpraktiker (BO 851), Physiotherapeutin und Physiotherapeut, Masseurin und Masseur, Medizinische(r) Bademeisterin und Bademeister (BO 852), Gesundheits- und Krankenpflegerin/-pfleger, Hebamme und Entbindungspfleger (BO 853), Gesundheits- und Krankenpflegehelferin/-helfer (BO 854), Diätassistentin und Diätassistent, Ernährungsfachleute (BO 855), Medizinische, Zahnmedizinische und Tiermedizinische Fachangestellte (BO 856), Medizinisch-technische(r) Assistentin und Assistent und verwandte Berufe (BO 857), Pharmazeutisch-technische(r) Assistentin und Assistent (BO 858), Therapeutische Berufe, a. n. g. (BO 859), Altenpflegerin und Altenpfleger (BO 864), Heilerziehungspflegerin und Heilerziehungspfleger (BO 866).

botsprojektion FIT) beziehungsweise 523 000 Erwerbstätige (Angebotsprojektion BIBB-DEMOS) erhöhen.

In Schaubild 4 wird berufliche Flexibilität bei der Angebotsprojektion einkalkuliert. Bezieht man auch Berufswechsler und Arbeitskräfte ohne Berufsausbildung mit in die Angebotsprojektion ein, so zeigt sich im Jahr 2005 ein Überangebot von 284 000 Erwerbspersonen im Berufsfeld 48. Ab 2018 zeichnet sich jedoch zunehmend ein Arbeitskräftemangel ab, der bis zum Jahr 2025 auf 255 000 Erwerbstätige (Angebotsprojektion FIT) bzw. 265 000 Erwerbstätige (Angebotsprojektion BIBB-DEMOS) ansteigt (siehe Schaubild 4).

Schaubild 4

Projektion der Erwerbstätigen und Erwerbspersonen im Berufsfeld 48[1)] mit beruflicher Flexibilität

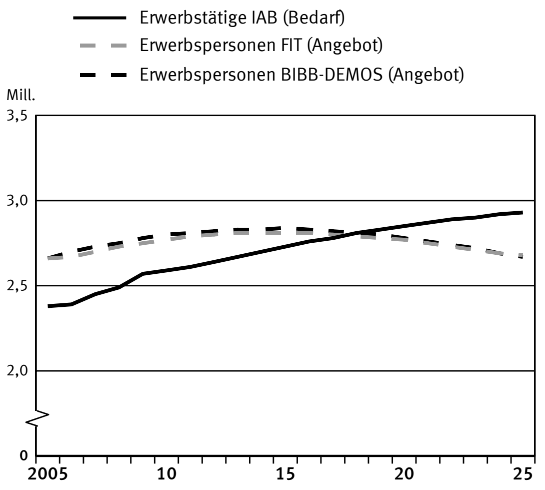

1) Gesundheitsberufe ohne Approbation.
Datenquelle: BIBB-IAB-Qualifikations- und Berufsfeldprojektionen, www.qube-projekt.de

2010 - 01 - 0815

2 Bedarfs- und Angebotsprojektionen für die Pflegeberufe

Im Abschnitt 1.3 wurde der Erwerbstätigenbedarf für das Berufsfeld 48 insgesamt anhand der bisherigen Entwicklung bis zum Jahr 2025 projiziert. Im Folgenden werden die Ergebnisse von Bedarfs- und Angebotsprojektionen für Pflegeberufe vorgestellt. Im Jahr 2005 übten 54,7 % der Erwerbstätigen aus dem Berufsfeld 48 einen Pflegeberuf aus (Ergebnis der Gesundheitspersonalrechnung). Zudem weisen Erwerbstätige in Pflegeberufen ähnliche berufliche Flexibilitäten auf wie Erwerbstätige im Berufsfeld 48 insgesamt (siehe Kapitel 3). Daher wird bei den Angebotsprojektionen die Entwicklung der Zahl der Erwerbstätigen und Erwerbspersonen aus dem Berufsfeld 48 auf die Pflegeberufe übertragen. Im Gegensatz zum vorhergehenden Abschnitt wird der Pflegepersonalbedarf jedoch nachfrageorientiert aus der bis zum Jahr 2025 zu erwartenden Zahl an Krankenhauspatientinnen und -patienten (Krankenhausfälle) und Pflegebedürftigen abgeleitet. Hierbei werden zwei Szenarien unterstellt: Einmal wird davon ausgegangen, dass die künftige Zahl der Krankenhausfälle sowie der Pflegebedürftigen allein von der Bevölkerungsentwicklung abhängig ist (Status-quo-Szenario). Das andere Szenario basiert auf der Überlegung, dass Menschen bei steigender Lebenserwartung verglichen mit heute erst in einem entsprechend späteren Lebensalter krank oder pflegebedürftig werden (Szenario „sinkender Behandlungsquoten“). Auch hier wird der ermittelte Bedarf den Angebotsprojektionen – mit und ohne Berücksichtigung der beruflichen Flexibilität – gegenübergestellt. Zudem werden bei der Angebotsprojektion auch unterschiedliche Beschäftigungsstrukturen berücksichtigt. Da sowohl das FIT-Modell wie auch das BIBB-DEMOS-Modell für das Berufsfeld 48 eine ähnliche Entwicklung aufzeigen, wird für die nachfolgenden Darstellungen der Einfachheit halber nur das BIBB-DEMOS-Modell abgebildet.

2.1 Abgrenzung der Pflegeberufe und Definition der betrachteten Größen

Zu den Pflegeberufen zählen „Gesundheits- und Krankenpflegerin/-pfleger“ [Berufsordnung (BO) 853], „Gesundheits- und Krankenpflegehelferin/-helfer“ (BO 854) sowie „Altenpflegerin/Altenpfleger“ (BO 864). Zur Gesundheits- und Krankenpflege gehört nach der Klassifikation der Berufe 1992 auch die Entbindungspflege. Im folgenden Text und in den folgenden Tabellen werden Hebammen und Entbindungspfleger nicht explizit aufgeführt, sondern sind in den Zahlen der Gesundheits- und Krankenpflegerinnen/-pfleger enthalten. Die Daten für Altenpflegerinnen und -pfleger umfassen auch Altenpflegehelferinnen und -helfer. Für diese drei Pflegeberufe werden im Rahmen der Gesundheitspersonalrechnung des Statistischen Bundesamtes unter anderem folgende Größen ermittelt und veröffentlicht: die Zahl der Beschäftigten, die Beschäftigungsart (Vollzeit, Teilzeit und geringfügige Beschäftigung), die Vollkräfte und die Einrichtungen, in denen die Beschäftigten in Pflegeberufen tätig sind. Die Zahl der *Beschäftigten in Pflegeberufen* basiert auf Beschäftigungsfällen, das heißt es werden alle Arbeitsverhältnisse berücksichtigt, unabhängig davon, ob es sich um Haupt- oder Nebenerwerbstätigkeiten handelt (im Jahr 2005 übten 98,3 % der Beschäftigten einen Pflegeberuf in ihrer Haupterwerbstätigkeit aus. Mehr als die Hälfte der nebenerwerbstätig Beschäftigten in Pflegeberufen waren in ihrer Haupterwerbstätigkeit ebenfalls als Pflegekraft tätig). Beschäftigte in Pflegeberufen mit zwei Arbeitsverträgen in unterschiedlichen Einrichtungen (zum Beispiel im Krankenhaus und im ambulanten Pflegedienst) werden daher doppelt gezählt. Das Erfassungskriterium bei der Gesundheitspersonalrechnung stellt nicht der berufliche Abschluss, sondern der aktuell ausgeübte Beruf dar. Daher sind in der Zahl der Beschäftigten in Pflegeberufen nicht nur *ausgebildete Pflegekräfte* enthalten – also Personen mit einem Berufsabschluss in einem der drei eingangs genannten Pflegeberufe –, sondern auch ungelernte oder berufsfremde Arbeitskräfte. Die Zahl der *Pflegevollkräfte* gibt die auf die volle tarifliche Arbeitszeit umgerechnete Zahl der Beschäftigten in Pflegeberufen an. Eine Pflegevollkraft entspricht dabei einer vollzeitbeschäftigten Person in Pflegeberufen.

Ob in Zukunft der Pflegepersonalbedarf durch das Angebot gedeckt werden kann, hängt neben der beruflichen Flexi-

bilität auch von der Beschäftigungsstruktur ab. Hiermit ist der Anteil der Vollzeit-, Teilzeit- und geringfügig Beschäftigten und deren geleistete Wochenarbeitszeit gemeint. Unterschiedliche Beschäftigungsstrukturen können durch Umrechnung in Pflegevollkräfte vergleichbar gemacht werden. Im Gegensatz zu den vorhergehenden Abschnitten sollen bei der Projektion des Pflegepersonalangebots und -bedarfs daher Vollkräfte statt Erwerbstätige und Erwerbspersonen betrachtet werden. Untersucht wird auch, wie stark sich die unterschiedlichen Beschäftigungsstrukturen von ost- und westdeutschen Beschäftigten in Pflegeberufen im Ausgangsjahr 2005 auf die Angebotsprojektionen der Pflegevollkräfte auswirken.

2.2 Beschäftigungsstruktur in Pflegeberufen im Ausgangsjahr 2005

Im Jahr 2005 übten rund 1,3 Millionen Beschäftigte einen Pflegeberuf aus (siehe Schaubild 5). Ein Großteil der Beschäftigten in Pflegeberufen war teilzeit- oder geringfügig beschäftigt (45,3 %). Daher lag die Zahl der Pflegevollkräfte mit 968 000 deutlich unter der Beschäftigtenzahl. Eine Vollzeitstelle in Pflegeberufen wurde im Jahr 2005 durchschnittlich von 1,3 Beschäftigten ausgefüllt.

Schaubild 5

Beschäftigte in Pflegeberufen nach Beschäftigungsart und Pflegevollkräfte 2005
Ergebnis der Gesundheitspersonalrechnung

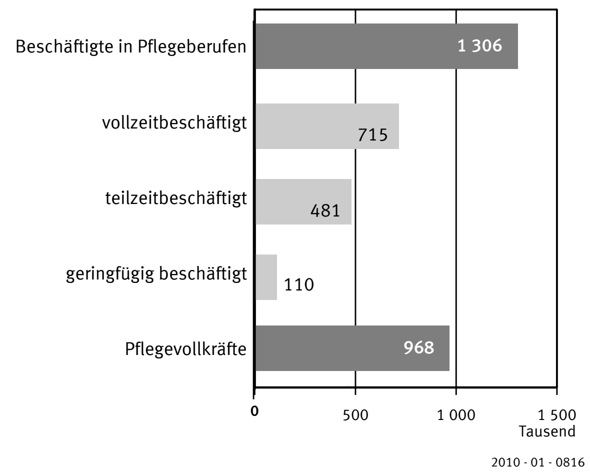

Der hohe Anteil an teilzeit- und geringfügig Beschäftigten ist vor allem auf den hohen Frauenanteil in den Pflegeberufen von insgesamt fast 84 % zurückzuführen (siehe Tabelle 1). Während 21,1 % der Männer in Pflegeberufen eine teilzeit- oder geringfügige Beschäftigung ausübten, war es bei den Frauen knapp die Hälfte (49,9 %). Zudem gibt es zwischen Ost und West große Unterschiede in der Beschäftigungsstruktur der weiblichen Beschäftigten in Pflegeberufen: Im Jahr 2005 gingen nach Ergebnissen des Mikrozensus in den neuen Ländern und Berlin-Ost 29,8 % mehr Frauen einer Vollzeitbeschäftigung in Pflegeberufen nach als im früheren Bundesgebiet. Außerdem arbeiteten die teilzeit- und geringfügig beschäftigten Frauen in Pflegeberufen im Osten durchschnittlich 4,9 Stunden beziehungsweise 4,7 Stunden je Woche länger als ihre westdeutschen Kolleginnen. Wiesen alle Beschäftigten in Pflegeberufen in Deutschland eine Beschäftigungsstruktur wie die in den neuen Ländern und Berlin-Ost auf, so würde die Zahl der Pflegevollkräfte um 9,5 % auf insgesamt rund 1,1 Millionen ansteigen. Hingegen würde die Zahl der Pflegevollkräfte bei einer auf ganz Deutschland übertragenen westdeutschen Beschäftigungsstruktur um 2,9 % auf 940 000 sinken.

Tabelle 1: Beschäftigte in Pflegeberufen 2005 nach Beruf
Ergebnis der Gesundheitspersonalrechnung

Berufsordnung	Beruf	Beschäftigte	Dar.: Frauen
		1 000	%
853	Gesundheits- und Krankenpflegerin/-pfleger	763	*86,4*
854	Gesundheits- und Krankenpflegehelferin/-helfer	232	*71,1*
864	Altenpflegerin/Altenpfleger	311	*87,4*
–	Pflegeberufe insgesamt	1 306	*83,9*

Auch im Hinblick auf die Gründe für die Teilzeitbeschäftigung (siehe Tabelle 2) unterscheiden sich die weiblichen Beschäftigten in Pflegeberufen im Osten deutlich von ihren westdeutschen Kolleginnen: Während die Beschäftigten in Pflegeberufen im Osten hauptsächlich teilzeitbeschäftigt waren, weil keine Vollzeitstelle zu finden war (46,2 %), dominierten im Westen deutlich persönliche oder familiäre Verpflichtungen (69,0 %).

Tabelle 2: Weibliche Teilzeitbeschäftigte 2005 in Pflegeberufen1)
Ergebnis des Mikrozensus
Prozent

Hauptgrund für Teilzeit	Neue Länder und Berlin-Ost	Früheres Bundesgebiet
Aufgrund von Krankheit, Unfallfolgen	/	2,8
Persönliche oder familiäre Verpflichtungen	31,0	69,0
Schulausbildung oder sonstige Aus- beziehungsweise Fortbildung	/	2,9
Vollzeittätigkeit nicht zu finden	46,2	11,5
Vollzeittätigkeit aus anderen Gründen nicht gewünscht	15,3	12,5

1) Die Angaben basieren auf allen weiblichen Teilzeit- oder geringfügig Beschäftigten in Pflegeberufen.

Beschäftigte in Pflegeberufen arbeiten überwiegend in Krankenhäusern, ambulanten oder (teil-)stationären Pflegeeinrichtungen. Im Jahr 2005 entfielen 76,3 % von allen Pflegevollkräften auf diese drei Einrichtungen (siehe Tabelle 3), die im Folgenden *Prognoseeinrichtungen* genannt werden. Für die zu erwartende Zahl der Krankenhausfälle und die durch ambulante und (teil-)stationäre Einrichtungen zu versorgende Zahl von Pflegebedürftigen wurden vom Statistischen Bundesamt zwei Prognosen – „Status-quo-Szenario“ und Szenario der „sinkenden Behandlungsquoten“ – erstellt und veröffentlicht.[8]) Beim „Status-quo-Szenario“ wird unterstellt, dass die Zahl der Krankenhausfälle sowie die Zahl der

8) Siehe hierzu Statistische Ämter des Bundes und der Länder (Herausgeber): „Demografischer Wandel in Deutschland, Heft 2: Auswirkungen auf Krankenhausbehandlungen und Pflegebedürftige im Bund und in den Ländern, Ausgabe 2010“.

Tabelle 3: Pflegevollkräfte 2005 nach Beruf und Einrichtung
Ergebnis der Gesundheitspersonalrechnung

Berufsordnung	Beruf	Pflegevollkräfte	Krankenhaus	Ambulante Pflege	(Teil-)Stationäre Pflege	Prognoseeinrichtungen insgesamt
		1 000	%			
853	Gesundheits- und Krankenpflegerin/-pfleger	558	*67,7*	*9,3*	*9,2*	*86,3*
854	Gesundheits- und Krankenpflegehelferin/-helfer	171	*20,7*	*3,4*	*8,3*	*32,4*
864	Altenpflegerin/Altenpfleger	239	*6,8*	*18,0*	*59,5*	*84,3*
–	Pflegeberufe insgesamt	968	*44,4*	*10,4*	*21,5*	*76,3*

Pflegebedürftigen heute und in Zukunft allein von der Bevölkerungsentwicklung abhängen: Für die Vorausberechnung der Zahl der Krankenhausfälle wurden die gegenwärtigen alters- und geschlechtsspezifischen Krankenhausdiagnosewahrscheinlichkeiten (in Fünfjahres-Altersgruppen) aus den Jahren 2006, 2007 und 2008 konstant gehalten und auf die vorausberechnete Bevölkerung (nach der 12. koordinierten Bevölkerungsvorausberechnung) angelegt. Analog wurde bei der Vorausberechnung der Zahl der Pflegebedürftigen vorgegangen: Hier wurden alters- und geschlechtsspezifische Pflegequoten aus den Jahren 2005 und 2007 auf die vorausberechnete Bevölkerung übertragen. Beim Szenario der „sinkenden Behandlungsquoten“ wird davon ausgegangen, dass Menschen bei einer steigenden Lebenserwartung länger gesund bleiben beziehungsweise erst in einem höheren Alter pflegebedürftig werden. Daher wurden die aktuellen alters- und geschlechtsspezifischen Krankenhausdiagnose- beziehungsweise Pflegewahrscheinlichkeiten entsprechend dem Anstieg der Lebenserwartung in höhere Altersgruppen verschoben.

2.3 Grundschema der Bedarfs- und Angebotsprojektionen von Pflegevollkräften in Prognoseeinrichtungen

Im Folgenden wird das Grundschema der Bedarfs- und Angebotsprojektionen der Pflegevollkräfte in Prognoseeinrichtungen dargestellt. Die zugrunde liegenden Datenquellen können dem auf dieser Seite unten platzierten Kasten entnommen werden.

Schaubild 6

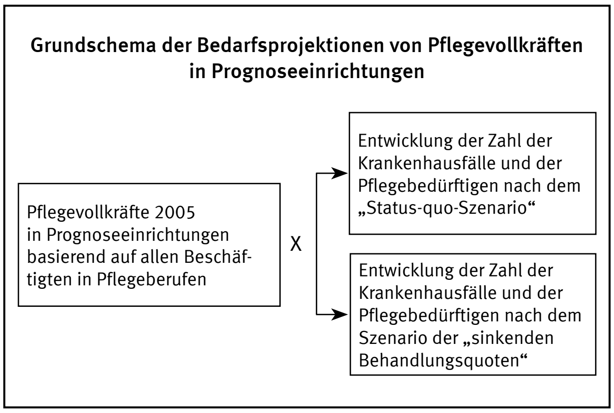

Die Ergebnisse der Bedarfsprojektionen nach dem „Status-quo-Szenario“ und dem Szenario der „sinkenden Behandlungsquoten“ sind in den Schaubildern 8 und 9 dargestellt. Bei den Bedarfsprojektionen wird über den gesamten Prognosezeitraum eine fixe Pflegepersonal-Patienten-Relation aus dem Jahr 2005 unterstellt. Dies ist nur dann gegeben,

Bedarfs- und Angebotsprojektionen der Pflegevollkräfte in Prognoseeinrichtungen mit und ohne Berücksichtigung der beruflichen Flexibilität

Bedarfsprojektion:

Die *Pflegevollkräfte 2005 in Prognoseeinrichtungen, basierend auf allen Beschäftigten in Pflegeberufen*, wurden innerhalb der Gesundheitspersonalrechnung ermittelt.

Die *Entwicklung der Zahl der Krankenhausfälle und der Pflegebedürftigen nach dem „Status-quo-Szenario“ und dem Szenario der „sinkenden Behandlungsquoten“* wurde durch eine Vorausberechnung der Krankenhausfälle und der Pflegebedürftigen durch das Statistische Bundesamt geschätzt.

Angebotsprojektion:

Die *Pflegevollkräfte 2005, basierend auf beschäftigten und erwerbslosen ausgebildeten Pflegekräften*, wurden auf Basis des Mikrozensus 2005 ermittelt: Zunächst wurden alle erwerbstätigen und erwerbslosen Personen im Alter ab 15 Jahren, die ihren höchsten beruflichen Abschluss im Berufsfeld 48 erworben haben (Fachkräfte im Berufsfeld 48), auf das Erwerbstätigenniveau der Volkswirtschaftlichen Gesamtrechnungen hochgerechnet. Anschließend erfolgte mithilfe des Mikrozensus und der Gesundheitspersonalrechnung eine Umrechnung der Fachkräfte im Berufsfeld 48 in Vollkräfte und Pflegevollkräfte. Zudem wurden die Pflegevollkräfte an das Niveau der Gesundheitspersonalrechnung angepasst.

Die *Abgänge in andere Berufe und Zugänge aus anderen Berufen* wurden ebenfalls auf Basis des Mikrozensus 2005 ermittelt, indem der ausgeübte Beruf dem höchsten beruflichen Abschluss gegenübergestellt wurde. Hierbei wurde das Maß an beruflicher Flexibilität aus dem Berufsfeld 48 auf die Pflegeberufe übertragen.

Die Pflegevollkräfte in Abhängigkeit von der *Beschäftigungsstruktur in Pflegeberufen wie in Deutschland/Ost/West 2005* wurden durch den Mikrozensus ermittelt und an das Niveau der Gesundheitspersonalrechnung angepasst.

Die *Angebotsentwicklung wie im Berufsfeld 48* wurde durch das BIBB-DEMOS-Modell (GWS) (siehe Kasten auf Seite 992) geschätzt und auf die Pflegeberufe übertragen.

Der Anteil der *Pflegevollkräfte in Prognoseeinrichtungen 2005* wurde durch die Gesundheitspersonalrechnung ermittelt.

Schaubild 7

Grundschema der Angebotsprojektionen von Pflegevollkräften in Prognoseeinrichtungen mit und ohne Berücksichtigung der beruflichen Flexibilität

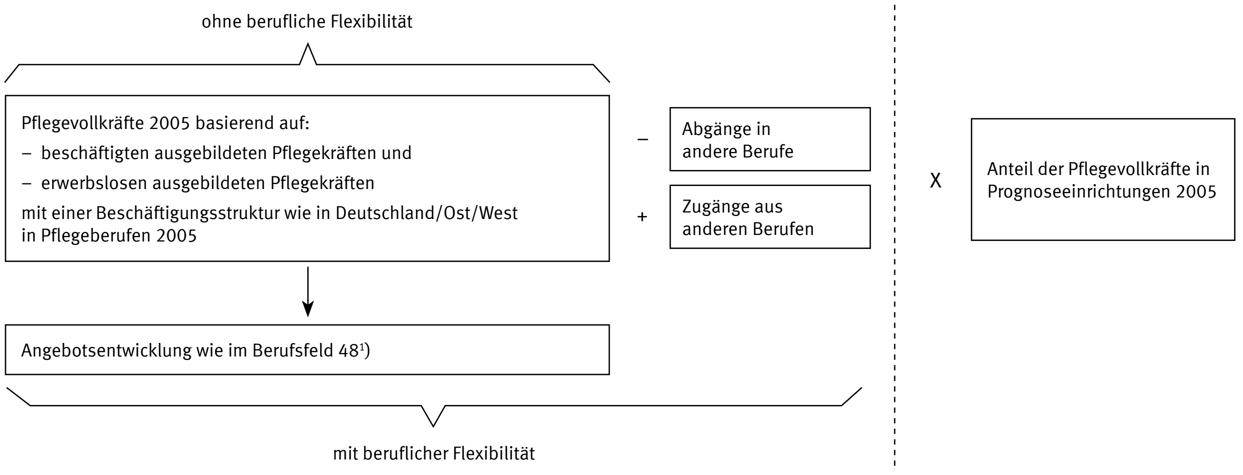

1) Gesundheitsberufe ohne Approbation.

wenn gleichzeitig insbesondere folgende Randbedingungen konstant bleiben:

- die durchschnittliche Verweildauer der Patientinnen und Patienten in Krankenhäusern,
- die Aufgabenteilung zwischen ärztlichem und nicht ärztlichem Personal,
- der Anteil der ausschließlich durch Angehörige gepflegten Personen sowie der Anteil der durch ambulante und (teil-)stationäre Pflegeeinrichtungen Versorgten,
- die Verteilung der Pflegebedürftigen auf die Pflegestufen I, II und III.

Die Ergebnisse der Angebotsprojektionen mit und ohne Berücksichtigung der beruflichen Flexibilität sind ebenfalls den Schaubildern zu entnehmen. Während die Pflegevollkräfte in den Angebotsprojektionen ohne berufliche Flexibilität (Schaubild 8) ausschließlich auf ausgebildeten Pflegekräften basieren, werden bei den Angebotsprojektionen mit beruflicher Flexibilität (Schaubild 9) auch un- und angelernte Pflegekräfte berücksichtigt.

Die Angebotsprojektionen mit und ohne Berücksichtigung der beruflichen Flexibilität fundieren auf folgenden methodischen Annahmen:

- Die berufliche Flexibilität aus dem Jahr 2005 bleibt über den gesamten Prognosezeitraum konstant.
- Gleiches Maß an beruflicher Flexibilität bei den Beschäftigten im Berufsfeld 48, den Beschäftigten in Pflegeberufen insgesamt und den Beschäftigten in Pflegeberufen in Prognoseeinrichtungen sowie den Beschäftigten in Haupt- und Nebenerwerbstätigkeit.
- Bezüglich der Beschäftigungsart (Vollzeit, Teilzeit und geringfügige Beschäftigung), der geleisteten Wochenarbeitszeit sowie des Anteils der Beschäftigten, die einen Pflegeberuf in einer Nebenerwerbstätigkeit ausüben, werden für die folgenden Gruppen gleiche Strukturen unterstellt:
  - ausgebildete Pflegekräfte und Beschäftigte in Pflegeberufen
  - Beschäftigte und Erwerbslose in Pflegeberufen
  - Beschäftigte in Pflegeberufen in Prognoseeinrichtungen und Beschäftigte in Pflegeberufen insgesamt
- Konstanter Anteil der Pflegevollkräfte in den drei Prognoseeinrichtungen: Der im Jahr 2005 ermittelte Anteil an Pflegevollkräften in den drei Prognoseeinrichtungen wird über den gesamten Prognosezeitraum konstant gehalten.

2.4 Ergebnisse der Bedarfs- und Angebotsprojektionen der Pflegevollkräfte in Prognoseeinrichtungen

Für die Jahre 2005 bis 2008 werden in den Bedarfskurven durch die Gesundheitspersonalrechnung ermittelte Pflegevollkräfte in den drei Prognoseeinrichtungen dargestellt. Die Bedarfsprognose beginnt mit dem Jahr 2009: Nach dem „Status-quo-Szenario“ steigt der Bedarf an Pflegevollkräften vom Ausgangsjahr 2005 bis zum Jahr 2025 in den drei Prognoseeinrichtungen insgesamt um 27,3 % an. Die kontinuierlich steigende Nachfrage nach Beschäftigten in Pflegeberufen entwickelt sich in den Prognoseeinrichtungen unterschiedlich stark: Während sich nach dem „Status-quo-Szenario“ die Zahl der Krankenhausfälle bis zum Jahr 2025 um 12,4 % erhöht, wächst die Zahl der Pflegebedürftigen und somit auch die Zahl der in ambulanten und (teil-)stationären Pflegeeinrichtungen benötigten Pflegevollkräfte um

Schaubild 8

Bedarfs- und Angebotsentwicklung an Pflegevollkräften in Prognoseeinrichtungen ohne berufliche Flexibilität

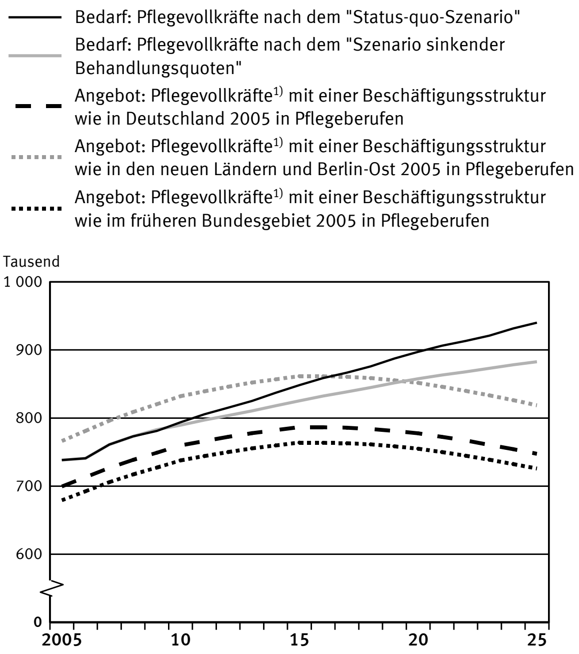

1) Personen mit höchstem Berufsabschluss in Pflegeberufen.

2010 - 01 - 0817

48,1 %. Nach dem Szenario der „sinkenden Behandlungsquoten“ steigt der Bedarf an Pflegevollkräften bis zum Jahr 2025 um insgesamt 19,5 %. Auch hier wächst der Bedarf in Krankenhäusern bis zum Jahr 2025 mit + 8,1 % schwächer als der in der ambulanten und (teil-)stationären Pflege mit einem Zuwachs von 35,4 %.

In den Angebotskurven liegen für das Ausgangsjahr 2005 auf Basis des Mikrozensus ermittelte empirische Werte vor. Für die Folgejahre wurde das Angebot an Pflegevollkräften für die Prognoseeinrichtungen mit dem Prognosemodell BIBB-DEMOS fortgeschrieben. Im Ausgangsjahr 2005 wurden in Schaubild 8 bei der Ermittlung der Pflegevollkräfte nur ausgebildete Pflegekräfte mit einem höchsten Berufsabschluss in Pflegeberufen berücksichtigt (siehe Schaubild 7). Unterschiedliche Ausgangswerte im Jahr 2005 und Endwerte im Jahr 2025 beruhen auf den unterschiedlichen Beschäftigungsstrukturen in Pflegeberufen für Gesamt-, Ost- und Westdeutschland und der hieraus resultierenden Zahl an Pflegevollkräften: Wäre in Pflegeberufen ausschließlich ausgebildetes Pflegepersonal beschäftigt, so fehlten bereits im Ausgangsjahr 2005 in Deutschland 39 000 Pflegevollkräfte. Wiesen die ausgebildeten Pflegekräfte in Gesamtdeutschland eine Beschäftigungsstruktur auf wie die westdeutschen Beschäftigten in Pflegeberufen, so würde der Mangel auf insgesamt 59 000 Pflegevollkräfte anwachsen. Ein Überangebot an Pflegevollkräften bestünde im Ausgangsjahr 2005 dann (+ 28 000), wenn alle ausgebildeten Pflegekräfte eine Beschäftigungsstruktur hätten wie die in den neuen Ländern und Berlin-Ost 2005. Hätten alle ausgebildeten Pflegekräfte über den gesamten Prognosezeitraum eine Beschäftigungsstruktur wie in Deutschland beziehungsweise im früheren Bundesgebiet 2005, so würde der Mangel an ausgebildeten Pflegekräften bis zum Jahr 2025 auf 193 000 beziehungsweise 214 000 Pflegevollkräfte („Status-quo-Szenario“) und auf 135 000 beziehungsweise 157 000 Pflegevollkräfte (Szenario „sinkender Behandlungsquoten“) anwachsen. Der Bedarf an ausgebildeten Pflegekräften könnte nach dem „Status-quo-Szenario“ bis zum Jahr 2016 und nach dem Szenario der „sinkenden Behandlungsquoten“ bis zum Jahr 2019 durch das Angebot gedeckt werden, wenn alle ausgebildeten Pflegekräfte über den gesamten Prognosezeitraum eine Beschäftigungsstruktur wie die im Osten aufwiesen. Aber auch in diesem Fall würde sich bis zum Jahr 2025 ein Mangel an ausgebildeten Pflegekräften von 121 000 Pflegevollkräften („Status-quo-Szenario“) beziehungsweise 64 000 Pflegevollkräften (Szenario „sinkender Behandlungsquoten“) einstellen.

Aus der Presse ist allgemein bekannt, dass es bereits zum aktuellen Zeitpunkt schwierig ist, neues Pflegepersonal einzustellen. Schaubild 8 bestätigt dieses Bild, da der Bedarf an ausgebildeten Pflegekräften nach der gesamtdeutschen Beschäftigungsstruktur bereits im Ausgangsjahr 2005 über dem Angebot liegt. Dieser Pflegepersonalmangel kann bisher aber noch durch die Beschäftigung un- und angelernter Pflegekräfte kompensiert werden. In Schaubild 9 ist die berufliche Flexibilität bei den Angebotsprojektionen einkalkuliert (siehe Schaubild 7). Bei der zusätzlichen Berücksichtigung un- und angelernter Pflegekräfte liegen alle drei

Schaubild 9

Bedarfs- und Angebotsentwicklung an Pflegevollkräften in Prognoseeinrichtungen mit beruflicher Flexibilität

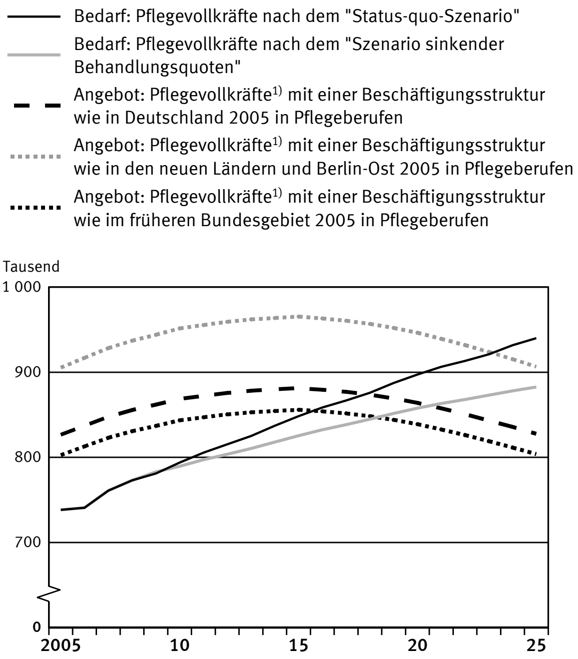

1) Basierend auf allen Beschäftigten in Pflegeberufen.

2010 - 01 - 0817

Angebotsausgangswerte im Jahr 2005 deutlich über dem Bedarf an Pflegevollkräften in den drei Prognoseeinrichtungen. Hätten alle Beschäftigten in Pflegeberufen künftig eine Beschäftigungsstruktur wie in Deutschland 2005 in Pflegeberufen, so träte ein Pflegepersonalmangel nach dem „Status-quo-Szenario“ ab 2018 und nach dem Szenario der „sinkenden Behandlungsquoten“ ab 2021 ein. Bis zum Jahr 2025 wüchse dieses Defizit dann auf 112 000 („Status-quo-Szenario“) beziehungsweise 55 000 Pflegevollkräfte (Szenario der„sinkenden Behandlungsquoten“) an. Bei einer westdeutschen Beschäftigungsstruktur würde der Pflegepersonalmangel bereits zwei Jahre früher eintreten. Ein ausreichendes Angebot an Pflegevollkräften läge bis zum Jahr 2025 nur dann vor (+ 24 000 Pflegevollkräfte), wenn sich die Zahl der Krankenhausfälle und der Pflegebedürftigen nach dem Szenario der „sinkenden Behandlungsquoten“ entwickelten und alle Beschäftigten in Pflegeberufen künftig eine Beschäftigungsstruktur aufwiesen wie die in den neuen Ländern und Berlin-Ost 2005. Nach dem „Status-quo-Szenario“ würde auch bei einer ostdeutschen Beschäftigungsstruktur in Pflegeberufen nach 2023 ein Pflegepersonalmangel auftreten, der bis 2025 auf 34 000 Pflegevollkräfte anwachsen würde.

3 Berufliche Flexibilität und Angebotspotenziale

Bei der Betrachtung von Schaubild 8 zeigt sich, dass der Bedarf an Pflegevollkräften bereits im Ausgangsjahr 2005 nicht allein durch ausgebildete Pflegekräfte gedeckt werden konnte. Stattdessen waren Zugänge von Beschäftigten aus anderen Berufen in dieses Berufsfeld notwendig, um den Bedarf an Pflegevollkräften zu decken. Schaubild 9 zeigt, dass durch die sogenannte berufliche Flexibilität im Jahr 2005 mehr potenzielle Pflegevollkräfte zur Verfügung standen, als benötigt wurden. Dennoch würde bei einem „Status-quo-Szenario“ und einer Beschäftigungsstruktur in Pflegeberufen wie in Gesamtdeutschland 2005 – auch unter Berücksichtigung von beruflicher Flexibilität – ab dem Jahr 2018 ein Beschäftigtenmangel im Bereich der Pflegeberufe eintreten.

Im Folgenden wird die berufliche Flexibilität in Pflegeberufen und im Berufsfeld 48 auf Basis des Mikrozensus 2005[9]) analysiert. Hierfür werden die beiden Betrachtungsebenen „Berufsordnung erlernter Beruf“ und „Berufsordnung ausgeübter Beruf“ herangezogen. Die Betrachtungsebene „Berufsordnung erlernter Beruf“ zeigt das Verhalten derjenigen, die einen Pflegeberuf erlernt haben. Hingegen wird auf der Betrachtungsebene „Berufsordnung ausgeübter Beruf“ dargestellt, wer in Pflegeberufen und im Berufsfeld 48 im Jahr 2005 arbeitet. Personen, die in ihrem erlernten Beruf verweilen, werden im Folgenden als *Stayer* bezeichnet; Personen, die ihren erlernten Beruf verlassen, als *Mover*.

Bei der dargestellten beruflichen Flexibilität in Schaubild 9 handelt es sich um dieselbe berufliche Flexibilität wie aus und in das Berufsfeld 48[10]) (siehe Schaubild 4). Die Flexibilisierungsvorgänge aus diesem Berufsfeld wurden auf die Pflegeberufe übertragen: Das heißt es wurde angenommen, dass sich Ab- und Zugänge aus und in dieses Berufsfeld gleichmäßig über die darin zusammengefassten Berufe verteilen. Im Folgenden wird untersucht, ob in Pflegeberufen die gleiche berufliche Flexibilität vorliegt wie im Berufsfeld 48: Tabelle 4 zeigt auf der Betrachtungsebene „Berufsordnung erlernter Beruf“, dass 75,4 % der Personen, die einen Beruf innerhalb des Berufsfeldes 48 erlernt haben, auch in diesem Berufsfeld arbeiten (Stayer im Berufsfeld 48). Die berufliche Flexibilität der ausgebildeten Pflegekräfte unterscheidet sich etwas von der Flexibilität der Fachkräfte aus dem übergeordneten Berufsfeld 48: Der Anteil der Stayer ist unter den ausgebildeten Pflegekräften insgesamt um 9,1 Prozentpunkte (74,8 % gegenüber 65,9 %) höher als unter den Fachkräften des Berufsfeldes 48 insgesamt. Dies bedeutet, dass ausgebildete Pflegekräfte ihren erlernten Beruf im Durchschnitt seltener verlassen als Fachkräfte im übergeordneten Berufsfeld 48.

Betrachtet man die berufliche Zusammensetzung der Beschäftigten in den Pflegeberufen („Berufsordnung ausgeübter Beruf“ in Tabelle 5), so stellt man fest, dass sich hier die Verteilung zwischen Ausgebildeten und An- bzw. Ungelernten in den Pflegeberufen nicht wesentlich von der Zusammensetzung der Beschäftigten innerhalb des Berufsfeldes 48 unterscheidet. Die ausgebildeten Pflegekräfte und die Fachkräfte aus dem Berufsfeld 48 verweilen jeweils zu 56,4 % beziehungsweise 57,1 % innerhalb der Berufsordnung und zu 64,5 % beziehungsweise 65,4 % innerhalb des Berufsfeldes.

Tabelle 4: Mover[1]) und Stayer[2]) in Pflegeberufen und im Berufsfeld 48 nach dem erlernten Beruf

Ergebnis des Mikrozensus 2005[3])

Berufsordnung, Berufsfeld	Erlernter Beruf	Fallzahl	Mover aus der Berufsordnung	Stayer in der Berufsordnung	Mover aus dem Berufsfeld	Stayer im Berufsfeld
		Anzahl	%			
853	Gesundheits- und Krankenpflegerin/-pfleger	6 182	*23,6*	*76,4*	*14,2*	*85,8*
854	Gesundheits- und Krankenpflegehelferin/-helfer	363	*50,1*	*49,9*	*30,3*	*69,7*
864	Altenpflegerin/Altenpfleger	1 623	*25,6*	*74,4*	*12,7*	*87,3*
–	Pflegeberufe insgesamt	8 168	*25,2*	*74,8*	*14,7*	*85,3*
48	Gesundheitsberufe ohne Approbation	16 999	*34,1*	*65,9*	*24,6*	*75,4*

1) Personen, die ihren erlernten Beruf verlassen. – 2) Personen, die in ihrem erlernten Beruf verweilen. – 3) Ausgewertet wurden Personen in ihrer Haupterwerbstätigkeit (gewichtete Daten).

9) Die Ergebnisse liegen auch für die Mikrozensen 2006 bis 2008 vor. Da für die Angebotsprojektionen der BIBB-IAB-Qualifikations- und Berufsfeldprojektionen aber das Jahr 2005 als Ausgangsjahr dient, wird der Mikrozensus 2005 als Datenbasis für die Erklärung der beruflichen Flexibilitäten verwendet. Die Ergebnisse unterscheiden sich jedoch nur geringfügig von denen der Mikrozensen der darauffolgenden Jahre.

10) Siehe hierzu Maier, T./Schandock, M./Zopf, S.: „Flexibilität zwischen erlerntem und ausgeübtem Beruf“ in Helmrich, R./Zika, G. (Fußnote 2), hier: Seite 153 ff.

Tabelle 5: Mover[1]) und Stayer[2]) in Pflegeberufen und im Berufsfeld 48 nach dem ausgeübten Beruf
Ergebnis des Mikrozensus 2005[3])

Berufsordnung, Berufsfeld	Ausgeübter Beruf	Fallzahl	Mover in die Berufsordnung	Stayer in der Berufsordnung	Mover in das Berufsfeld	Stayer im Berufsfeld
		Anzahl	%			
853	Gesundheits- und Krankenpflegerin/-pfleger	6 255	24,9	75,1	19,4	80,6
854	Gesundheits- und Krankenpflegehelferin/-helfer	1 274	85,3	14,7	70,6	29,4
864	Altenpflegerin/Altenpfleger	3 222	62,8	37,2	52,5	47,5
–	Pflegeberufe insgesamt	10 751	43,6	56,4	35,5	64,5
48	Gesundheitsberufe ohne Approbation	19 577	42,9	57,1	34,6	65,4

1) Personen, die ihren erlernten Beruf verlassen. – 2) Personen, die in ihrem erlernten Beruf verweilen. – 3) Ausgewertet wurden Personen in ihrer Haupterwerbstätigkeit (gewichtete Daten).

Wenn die berufliche Zusammensetzung der Beschäftigten in den Pflegeberufen gleich der beruflichen Zusammensetzung der Beschäftigten des Berufsfeldes 48 ist, jedoch mehr ausgebildete Pflegekräfte in ihrem erlernten Beruf verweilen als Fachkräfte insgesamt in Berufsfeld 48, dann bedeutet das für das Angebot an Pflegevollkräften, dass dies auch etwas höher ausfallen kann als in Schaubild 9 dargestellt. Denn wenn mehr ausgebildete Pflegekräfte in ihrem erlernten Beruf verweilen, müssen auch mehr An- bzw. Ungelernte in die Pflegeberufe zuwandern, damit die berufliche Verteilung innerhalb der Beschäftigten in den Pflegeberufen gleich der beruflichen Verteilung des Berufsfeldes 48 ist. Die Pflegeberufe scheinen also von der beruflichen Flexibilität etwas mehr zu profitieren als das Berufsfeld 48 insgesamt. Es ist denkbar, dass die drei unterschiedlichen Angebotsprojektionen an Pflegevollkräften in Prognoseeinrichtungen unter Berücksichtigung der beruflichen Flexibilität jeweils etwas höher ausfallen als in Schaubild 9 abgebildet.

Wenn 74,8 % der ausgebildeten Pflegekräfte nur 56,4 % der Beschäftigten in Pflegeberufen stellen, wird noch einmal verdeutlicht, dass der Pflegepersonalbedarf nicht allein über die ausgebildeten Pflegekräfte gedeckt werden kann. Innerhalb der einzelnen Pflegeberufe ergeben sich allerdings Unterschiede in der Flexibilität: Bei den Gesundheits- und Krankenpflegerinnen/-pflegern können drei Viertel des Bedarfs durch ausgebildete Pflegekräfte gedeckt werden, weitere 5,5 % rekrutieren sich aus den verwandten Berufen innerhalb desselben Berufsfeldes. Anders verhält es sich bei den Altenpflegerinnen und Altenpflegern. Obwohl hier 74,4 % in ihrem erlernten Berufen arbeiten, stellen sie lediglich 37,2 % der als Altenpflegerinnen und Altenpfleger Beschäftigten. Weitere 10,3 % der als Altenpflegerinnen und Altenpfleger Beschäftigten stammen aus den verwandten Berufen desselben Berufsfeldes. Ein höherer Bedarf beziehungsweise eine größere Flexibilität herrscht bei den Gesundheits- und Krankenpflegehelferinnen/-helfern vor. Sie stellen nur 14,7 % der als Gesundheits- und Krankenpflegehelferinnen/-helfer Beschäftigten. Diese 14,7 % sind 49,9 % derer, die diesen Beruf erlernt haben. Gesundheits- und Krankenpflegehelferinnen/-helfer scheinen die flexibelsten unter den Pflegeberufen zu sein, während die

Tabelle 6: Mover[1]) und Stayer[2]) in Pflegeberufen und im Berufsfeld 48 nach Beschäftigungsart
Ergebnis des Mikrozensus 2005[3])

Berufsordnung, Berufsfeld	Beruf	Fallzahl	Mover aus der Berufsordnung	Stayer in der Berufsordnung	Mover aus dem Berufsfeld	Stayer im Berufsfeld
		Anzahl	%			
		Vollzeit[4])				
853	Gesundheits- und Krankenpflegerin/-pfleger	3 723	20,5	79,5	11,8	88,2
854	Gesundheits- und Krankenpflegehelferin/-helfer	250	47,6	52,4	29,1	70,9
864	Altenpflegerin/Altenpfleger	1 011	23,8	76,2	10,6	89,4
–	Pflegeberufe insgesamt	4 984	22,6	77,4	12,4	87,6
48	Gesundheitsberufe ohne Approbation	9 865	31,4	68,6	22,1	77,9
		Teilzeit				
853	Gesundheits- und Krankenpflegerin/-pfleger	1 946	21,9	78,1	12,3	87,7
854	Gesundheits- und Krankenpflegehelferin/-helfer	81	/	/	/	/
864	Altenpflegerin/Altenpfleger	496	24,5	75,5	11,7	88,3
–	Pflegeberufe insgesamt	2 523	23,4	76,6	12,7	87,3
48	Gesundheitsberufe ohne Approbation	5 177	32,6	67,4	22,5	77,5
		geringfügig beschäftigt[5])				
853	Gesundheits- und Krankenpflegerin/-pfleger	513	52,2	47,8	39,3	60,7
854	Gesundheits- und Krankenpflegehelferin/-helfer	/	/	/	/	/
864	Altenpflegerin/Altenpfleger	116	47,1	52,9	/	/
–	Pflegeberufe insgesamt	661	52,1	47,9	39,3	60,7
48	Gesundheitsberufe ohne Approbation	1 895	51,3	48,7	42,6	57,4

1) Personen, die ihren erlernten Beruf verlassen. – 2) Personen, die in ihrem erlernten Beruf verweilen. – 3) Ausgewertet wurden Personen in ihrer Haupterwerbstätigkeit (gewichtete Daten). – 4) Personen mit einer normalerweise geleisteten Arbeitszeit von mehr als 31 Stunden werden im Mikrozensus grundsätzlich als Vollzeitbeschäftigte gezählt. – 5) Es werden alle Befragten als „geringfügig beschäftigt“ eingestuft, bei denen die geringfügige Beschäftigung die einzige oder hauptsächliche Tätigkeit darstellt.

Gesundheits- und Krankenpflegerinnen/-pfleger und Altenpflegerinnen und Altenpfleger relativ starr in ihrer Berufsausübung sind. Der Anteil an Stayern (76,4 % beziehungsweise 74,4 %) liegt hier jeweils über dem durchschnittlichen Anteil der Stayer in den Berufsordnungen (65,9 %) innerhalb des Berufsfeldes 48. Trotz der geringen Abwanderung aus diesen Berufen in fachfremde Berufe gelang es jedoch bereits im Jahr 2005 nicht, den Bedarf an Beschäftigten in Pflegeberufen allein aus diesen Berufen beziehungsweise aus verwandten Berufen innerhalb des Berufsfeldes zu decken. Stattdessen mussten beispielsweise 52,5 % der Altenpflegerinnen und Altenpfleger aus fachfremden Berufen (außerhalb des Berufsfeldes 48 „Gesundheitsberufe ohne Approbation") gewonnen oder der Bedarf durch Auszubildende oder Personen ohne formalen beruflichen Abschluss gedeckt werden.

Schaubild 9 zeigt auch, dass ein Engpass an Beschäftigten in Pflegeberufen erst ab dem Jahr 2023 eintritt, sofern sich die derzeitige westdeutsche Beschäftigungsstruktur der ostdeutschen Beschäftigungsstruktur anpasst. Ein solches Szenario erscheint aber nur plausibel, wenn Vollzeit- und Teilzeitkräfte dieselben Flexibilitäten aufweisen. Aus Tabelle 6 ist ersichtlich, dass dies der Fall ist. So sind die Stayer- und Mover-Anteile zwischen Vollzeit- und Teilzeitbeschäftigten in allen Pflegeberufen ähnlich verteilt. Dies lässt den Schluss zu, dass eine Umwandlung von Teilzeit- in Vollzeitstellen (aber auch umgekehrt) vermutlich keine starken Effekte auf die berufliche Flexibilität der ausgebildeten Pflegekräfte hat. Lediglich ausgebildete Pflegekräfte in geringfügigen Beschäftigungsverhältnissen scheinen eher in fachfremden Berufen zu arbeiten als ihre vollzeit- beziehungsweise teilzeitbeschäftigten Kolleginnen und Kollegen: Der Stayer-Anteil der ausgebildeten Pflegekräfte insgesamt in geringfügigen Beschäftigungsverhältnissen liegt um 28,7 beziehungsweise 29,5 Prozentpunkte unter dem Stayer-Anteil der Teilzeit- beziehungsweise Vollzeitkräfte. Betrachtet man den Stayer-Anteil im Berufsfeld, sind es 26,6 bzw. 26,9 Prozentpunkte. Es wäre an dieser Stelle durchaus denkbar, dass bei einer Umwandlung von geringfügigen Beschäftigungsverhältnissen in Vollzeit- oder Teilzeitbeschäftigungen ausgebildete Pflegekräfte eher wieder bereit sind, in ihrem erlernten Beruf zu arbeiten.

4 Schlussbetrachtung

Aufgrund des demografischen Wandels wird der deutsche Arbeitsmarkt spätestens nach 2025 mit einem massiven Arbeitskräftemangel konfrontiert (siehe Schaubild 1). In den „Gesundheitsberufen ohne Approbation" wird dieser Mangel schon ab 2018 eintreten (siehe Schaubild 4). Auch der Bedarf an Pflegepersonal, das Teil dieses Berufsfeldes ist, kann bei einer Fortschreibung der derzeitigen alters- und geschlechtsspezifischen Krankenhausdiagnose- und Pflegewahrscheinlichkeiten (Status-quo-Szenario) und unter Beibehaltung der derzeitigen Beschäftigtenstruktur zu diesem Zeitpunkt nicht mehr gedeckt werden (siehe Schaubild 8). Bereits jetzt müssen fachfremde Arbeitskräfte um- oder angelernt werden, um ausgebildete Pflegekräfte zu ersetzen oder um diese in ihren Aufgaben zu unterstützen (siehe Schaubild 9).

Neben dem Aufzeigen von Engpässen werden in diesem Beitrag aber auch Lösungswege genannt, wie der Herausforderung eines Pflegepersonalmangels begegnet werden kann. Die „Stellschrauben" sind zum einen in der beruflichen Flexibilität, zum anderen in der Beschäftigungsstruktur in Pflegeberufen zu finden:

Es zeigt sich, dass ausgebildete Pflegekräfte zu hohen Anteilen in ihrem erlernten Beruf arbeiten. Wenn junge Menschen für eine Ausbildung in Pflegeberufen gewonnen werden können, dann werden diese bei einer dauerhaften Erwerbstätigkeit auch ihrem Ausbildungsberuf treu bleiben. Der hohe Anteil der An- oder Ungelernten, insbesondere unter den Gesundheits- und Krankenpflegehelferinnen/-helfern und den Altenpflegerinnen und Altenpflegern zeigt aber auch, dass eine Zuwanderung in diese Berufe offensichtlich möglich ist. Dies setzt aber auch eine gewisse Attraktivität der Pflegeberufe im Vergleich zu anderen Berufen voraus, für welche sich die fachfremden Arbeitskräfte entscheiden könnten. Gerade die Attraktivität eines Berufes wird in Zukunft an Bedeutung gewinnen, da Erwerbspersonen aufgrund des zunehmenden Arbeitskräftemangels freier zwischen verschiedenen Berufen werden wählen können. Dabei spielt neben der Bezahlung unter anderem auch die Beschäftigungsart eine Rolle. Tabelle 6 zeigt, dass der Anteil der ausgebildeten Pflegekräfte unter den Vollzeit- beziehungsweise Teilzeitbeschäftigten höher ist als unter den geringfügig Beschäftigten. Eine reine Aufstockung der Anzahl der Beschäftigten in Pflegeberufen über geringfügige Beschäftigungsverhältnisse würde dem Mangel an ausgebildeten Pflegekräften deshalb nicht begegnen. Denn ausgebildete Pflegekräfte arbeiten eher als Teilzeit- beziehungsweise als Vollzeitkräfte.

Eine Umwandlung von geringfügigen Beschäftigungsverhältnissen in Teilzeitverhältnisse oder der Teilzeit- in Vollzeitverhältnisse beziehungsweise eine Anhebung der geleisteten Wochenstunden bei Teilzeit- oder geringfügig Beschäftigten würde den Pflegepersonalengpass verzögern. So zeigt das Szenario der ostdeutschen Beschäftigtenstruktur, dass ein rechnerischer Mangel bei einem Status-quo-Szenario rund fünf Jahre später auftritt. Bei einem Szenario der „sinkenden Behandlungsquoten" liegt der rechnerische Engpass sogar außerhalb des Prognosezeitraums (siehe Schaubild 9). Bei einem Rückblick auf die Entwicklung der Beschäftigungsverhältnisse seit Beginn des Jahrtausends lässt sich in der Gesundheitspersonalrechnung allerdings kein Trend für eine steigende Anzahl an Vollzeitbeschäftigungsverhältnissen erkennen. Stattdessen gewinnen Teilzeit- und geringfügige Beschäftigungsverhältnisse immer mehr an Bedeutung. Ein Hauptgrund für Teilzeit- und geringfügige Beschäftigungen der größtenteils weiblichen Beschäftigten in Pflegeberufen scheinen insbesondere im früheren Bundesgebiet „persönliche oder familiäre Verpflichtungen" zu sein (siehe Tabelle 2). Dies bedeutet, dass eine Aufstockung der Arbeitszeit für einen Teil der Beschäftigten auch dann nicht möglich wäre, wenn die Prognoseeinrichtungen dies anbieten würden. Ein Grund hierfür könnte sein, dass sich Fami-

lie und Beruf für das weibliche Pflegepersonal teilweise nur schwer miteinander vereinbaren lassen. Dabei könnte eine bessere Vereinbarkeit von Familie und Beruf in den Pflegeberufen auch dazu beitragen, die Attraktivität der Pflegeberufe für junge Frauen zu steigern.

Auszug aus Wirtschaft und Statistik

© Statistisches Bundesamt, Wiesbaden 2010
Vervielfältigung und Verbreitung, auch auszugsweise, mit Quellenangabe gestattet.

Herausgeber: Statistisches Bundesamt, Wiesbaden

Schriftleitung: Roderich Egeler
Präsident des Statistischen Bundesamtes
Verantwortlich für den Inhalt:
Brigitte Reimann,
65180 Wiesbaden
Telefon: +49 (0) 6 11/75 20 86

Internet: www.destatis.de

Ihr Kontakt zu uns: www.destatis.de/kontakt

Statistischer Informationsservice
Telefon: +49 (0) 6 11/75 24 05
Telefax: +49 (0) 6 11/75 33 30

Vertriebspartner: HGV Hanseatische Gesellschaft für Verlagsservice mbH
Servicecenter Fachverlage
Postfach 11 64
D-72125 Kusterdingen
Telefon: +49 (0) 70 71/93 53 50
Telefax: +49 (0) 70 71/93 53 35
destatis@s-f-g.com
www.destatis.de/publikationen

Erscheinungsfolge: monatlich
